# Brain Functional Connectivity in Low- and High-Grade Gliomas: Differences in Network Dynamics Associated with Tumor Grade and Location

**DOI:** 10.3390/cancers14143327

**Published:** 2022-07-08

**Authors:** Luca Pasquini, Mehrnaz Jenabi, Onur Yildirim, Patrick Silveira, Kyung K. Peck, Andrei I. Holodny

**Affiliations:** 1 Neuroradiology Service, Department of Radiology, Memorial Sloan Kettering Cancer Center, New York, NY 10065, USA; jenabim@mskcc.org (M.J.); yildirio@mskcc.org (O.Y.); peckk@mskcc.org (K.K.P.); holodnya@mskcc.org (A.I.H.); 2 Neuroradiology Unit, NESMOS Department, Sant’Andrea Hospital, La Sapienza University, 00189 Rome, Italy; 3 Molecular Imaging and Therapy Service, Department of Radiology, Memorial Sloan Kettering Cancer Center, New York, NY 10065, USA; silveirp@mskcc.org; 4 Department of Medical Physics, Memorial Sloan Kettering Cancer Center, New York, NY 10065, USA; 5 Brain Tumor Center, Memorial Sloan Kettering Cancer Center, New York, NY 10065, USA; 6 Department of Radiology, Weill Medical College of Cornell University, New York, NY 10065, USA; 7 Department of Neuroscience, Weill-Cornell Graduate School of the Medical Sciences, New York, NY 10065, USA

**Keywords:** glioma, reorganization, fMRI, resting-state, plasticity, LGG, HGG, brain connectivity

## Abstract

**Simple Summary:**

This study investigates brain network modifications related to tumor grade and location using resting-state functional magnetic resonance imaging and graph theory. We demonstrated that low-grade gliomas (LGG) lead to increased efficiency of the surrounding functional network, while high-grade gliomas (HGG) seem to disrupt brain connectivity in remote areas. Tumor location appears to influence the pattern of reorganization, including the recruitment of the contralateral hemisphere. Overall, LGG may show more favorable connectivity changes than HGG. If confirmed by future studies, the ability to discriminate between ‘maladaptive’ (detrimental) and ‘adaptive’ (beneficial) functional reorganization based on graph theory metrics may provide biomarkers to select patients for surgery and monitor recovery.

**Abstract:**

Brain tumors lead to modifications of brain networks. Graph theory plays an important role in clarifying the principles of brain connectivity. Our objective was to investigate network modifications related to tumor grade and location using resting-state functional magnetic resonance imaging (fMRI) and graph theory. We retrospectively studied 30 low-grade (LGG), 30 high-grade (HGG) left-hemispheric glioma patients and 20 healthy controls (HC) with rs-fMRI. Tumor location was labeled as: frontal, temporal, parietal, insular or occipital. We collected patients’ clinical data from records. We analyzed whole-brain and hemispheric networks in all patients and HC. Subsequently, we studied lobar networks in subgroups of patients divided by tumor location. Seven graph-theoretical metrics were calculated (FDR *p* < 0.05). Connectograms were computed for significant nodes. The two-tailed Student t-test or Mann–Whitney U-test (*p* < 0.05) were used to compare graph metrics and clinical data. The hemispheric network analysis showed increased ipsilateral connectivity for LGG (global efficiency *p* = 0.03) and decreased contralateral connectivity for HGG (degree/cost *p* = 0.028). Frontal and temporal tumors showed bilateral modifications; parietal and insular tumors showed only local effects. Temporal tumors led to a bilateral decrease in all graph metrics. Tumor grade and location influence the pattern of network reorganization. LGG may show more favorable network changes than HGG, reflecting fewer clinical deficits.

## 1. Introduction

Brain tumors are disruptive lesions that lead to functional modifications of brain networks, known as functional reorganization or plasticity [1]. Short- and long-range effects have been shown using task-based and resting-state fMRI in patients with gliomas [2], possibly representing an adaptive phenomenon to compensate for tumor-induced clinical deficits. A better understanding of functional reorganization could optimize neurosurgical resection of brain tumors and tailor targeted therapies that enhance recovery [3]. Conversely, functional modifications induced by focal lesions may fail to compensate for lost functions or may even be detrimental. For example, right-hemispheric activation in left-sided stroke patients has been associated with failure of perilesional reorganization and a worse outcome [4]. When a focal lesion invades eloquent brain areas, the damage can trigger disinhibition of nearby neural networks, creating a permissive environment that may lead to compensatory (adaptive) or detrimental (maladaptive) plasticity [5]. Knowledge of plasticity dynamics and causative factors is lacking in the literature, despite being necessary for the highlighting of candidates for reorganization and also for the distinguishing of adaptive from maladaptive plasticity, which is a prerequisite for clinical implementation. Tumor pathology and location likely influence the network dynamics and may help to distinguish different types of plasticity. Classically, plastic changes have been described in low-grade gliomas (LGG) with a progressive timeline [6] from intra-tumoral and perilesional reorganization to activation of contralateral homologues. Some evidence supports the compensatory nature of plastic changes in LGG [7]. On the other hand, reorganization in high-grade gliomas (HGG) has not yet been demonstrated to be clinically meaningful. The location of a lesion may directly influence the magnitude of functional changes. For example, damage to brain regions important for communication between subnetworks (connectors) causes greater effects than damage to peripheral areas [8]. According to this model, damage to specific brain areas should lead to greater network changes.

Graph theory is applied to fMRI to study the architecture and complexity of functional networks [9,10] with relevant clinical applications [11]. Newly diagnosed gliomas demonstrated globally altered functional connectomic profiles in previous studies [12], while distributed changes of the functional connectome have been shown in glioblastoma patients after surgery [13]. Graph theory metrics can describe how efficient, integrated and connected a network is, quantifying properties such as small-worldness, expression of efficient information segregation and integration at low wiring and energy costs [14] (Supplementary Table S1).

Starting from this background, we studied the network modifications of left-hemispheric LGG and HGG versus healthy controls (HC) by applying graph theory to resting-state fMRI, with the following objectives: (1) compare the network modifications induced by tumors of different grades with respect to HC; (2) compare left vs. right-hemispheric network changes; (3) compare the network modifications induced by tumors of different locations with respect to HC. We selected only left-hemispheric tumors to be able to study ipsilateral and contralateral tumor effects in a uniform population. We investigated patients’ language performance as an example of left-lateralized function through the Boston Naming Test and neurologic/neurosurgical assessment of aphasia to identify possible clinical correlates of network changes. We hypothesized that tumors of different grades and locations would demonstrate characteristic intra-hemispheric and interhemispheric functional connectivity changes. Particularly, we hypothesized that HGG would display predominant regional modifications due to neurovascular uncoupling, while LGG would demonstrate both ipsi- and contralateral changes suggestive of reorganization. We also hypothesized LGG networks to be more integrated and connected than those of HGG, possibly reflecting better clinical performance.

## 2. Materials and Methods

### 2.1. Patients

This retrospective cross-sectional study was approved by the institutional review board and conducted in agreement with the Helsinki declaration. Informed consent was waived due to the retrospective design. We reviewed the archive of our institution from January 2016 to September 2021 to select patients with the following inclusion criteria: newly diagnosed left-hemispheric glioma (World Health Organization 2016 classification [15]); no prior surgery or other treatment; resting-state data acquired with the same protocol and therefore comparable; absence of tumor-related or patient-related artifacts including drop-out from hemorrhagic tumor components and motion. We applied these inclusion criteria to increment the homogeneity of our groups, excluding non-glioma tumors, and to limit the effect of susceptibility (from prior surgery or hemorrhagic tumor components) and motion artifacts on fMRI and brain connectivity measures. Twenty HC were included for comparison. Tumors were labeled according to their location in the brain (frontal, temporal, parietal, insular, occipital). Patients underwent presurgical cognitive testing with the Boston Naming Test [16]. Additionally, we reviewed the clinical records of our patients and collected clinical data regarding the presence of aphasia on neurologic and/or neurosurgical examinations performed before surgery and three months after surgery.

### 2.2. MRI Acquisition

MRI was performed using a 3T scanner (Discovery 750 W, GE Healthcare, Milwaukee, WI, USA) with 24-channel head coils. Resting-state fMRI was acquired with single-shot gradient echo EPI (TR/TE = 2500/32 ms, section thickness = 4 mm, matrix = 64 × 64, FOV = 240 mm, acquisition volume = 160, scanning duration = 6 min 55 s). fMRI coverage matching anatomical scans, including FLAIR (TR/TE = 10,000/106 ms, TI = 220 ms, matrix 256 × 256), T1 postcontrast (TR/TE = 600/20 ms, matrix 256 × 256), and 3D T1-weighted anatomic images using a spoiled gradient recalled-echo sequence (SPGR) (TR/TE = 22/4 ms, matrix 256 × 256, section thickness = 1 mm), were acquired as routine clinical scans. During the fMRI scan, subjects were instructed to relax, fixate on a central cross and try clear their mind clear during the scan. BrainWave RT (Medical Numerics), a real-time software on the GE scanners, was applied for quality control, including artifactual signal fluctuation and head motion. 

### 2.3. Functional Connectivity Analysis

**Figure 1:**
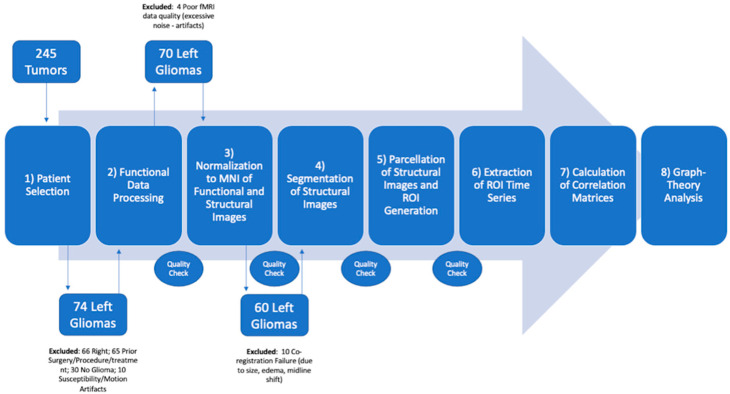
Schematic representation of the pipeline used for patient recruitment, data processing and analysis.

Pre- and post-processing of functional and structural data was performed through the CONN toolbox [17], implemented in SPM (SPM 12, The Wellcome Centre for Human Neuroimaging, UCL Queen Square Institute of Neurology, London, UK) and MATLAB (R2021b (9.11), The MathWorks Inc., Natick, MA, USA) packages. The default pre-processing for all patients included: functional realignment and estimation of motion parameters; slice-timing correction; outlier detection and head motion correction; direct normalization of functional and structural data to MNI space plus segmentation of all data to gray matter, white matter and cerebrospinal fluid; functional smoothing with a full-width-half-maximum (FWHM) of 6 mm; denoising with linear regression and temporal band pass filtering between 0.01 and 0.1 Hz; detrending. For denoising, CSF and WM nuisance regression were also included. The co-registered anatomical and functional images in MNI space were parcellated into 136 regions of interest (ROI) (136 parcels) using automated anatomical labeling—AAL atlas (the default atlas of CONN toolbox). All co-registered anatomical and functional images were inspected for the transformation of the tumor area to MNI space to ensure correct anatomical parcellation via the atlas. All the steps of our pipeline were monitored by two board-certified neuroradiologists to confirm the correct transformation to the standard space, correct segmentations and correct ROI fitting. Any necessary adjustments were carried out before proceeding to the following steps. A summary of the pipeline used for this study is presented in Figure 1. 

Finally, the average timeseries within each ROI were estimated. ROI-to-ROI connectivity matrices represent the connectivity between one of the 136 ROIs to the remaining ROIs. These matrices show the degree of functional connectivity between each pair of ROIs, with each element defined as the Fisher-transformed bivariate correlation coefficient between a pair of ROIs BOLD timeseries [17]. ROI-to-ROI connectivity matrices were obtained for three conditions of whole-brain ROIs, only left and only right hemisphere ROIs for each HGG, LGG and HC group. Graph theory analyses on each ROI-to-ROI matrix were conducted using the Louvain Algorithm in CONN toolbox. All ROI-level graph measures are based on nondirectional graphs with nodes (ROIs) and edges (supra-threshold connections). For each patient, a threshold of z > 2 and *p* < 0.05 was set to compute a graph adjacency matrix by thresholding the associated ROI-to-ROI matrix. From the resulting graphs, we then computed graph-theoretical measures to address the topological properties of each ROI within the graph, as well as of the entire network of ROIs [14]. Seven graph-theoretical metrics were calculated applying two-sided FDR correction (*p* < 0.05): global/local efficiency (representing the global and local connectivity of each ROI), betweenness centrality (showing the ratio of a time that a node is part of the shortest path between any two pairs of nodes in a graph), cost (proportion of edges from a node), average path length (the shortest distance between the current node and all other nodes), clustering coefficient (ratio of connected nodes to all neighboring nodes) and degree (the number of nodes to which the selected node was connected). A detailed description of these metrics is available elsewhere [14] and summarized in Supplementary Table S1. We used the above graph-theoretical metrics to evaluate the connectedness and integration of every node in the regional (local) and global networks. We considered the small-worldness of functional graphs to be the combination of high local clustering and short path length as well as high global/local efficiency and low cost [14]. 

### 2.4. Statistical Analyses

The Jarque–Bera test with chi-squared distribution and two degrees of freedom was used to confirm the normality of our data before applying any statistical comparisons [18]. The two-tailed Student *t*-test (in case of normal distribution) or Mann–Whitney U-test (in case of non-normal distribution) was employed to compare graph-theoretical metrics (*p* < 0.05) in the study groups for the global whole-brain network, left and right hemispheric networks. The first analysis was conducted with averaged graph-theoretical measures for each network (whole-brain and hemispheric network analysis). To account for tumor location effects, another analysis was repeated for subgroups of patients divided by location labels (lobar network analysis): for patients with a specific tumor location, we evaluated the nodes belonging to that location label in the whole-brain network. For example, nodes belonging to the frontal lobe were compared in patients with frontal lobe LGG, HGG and HC. Seed-to-ROI connectivity diagrams (connectograms) were generated on CONN by applying two-sided multi-comparison correction (FDR, *p* < 0.05). We used significant areas from prior analyses as seeds, while source ROIs from the connectivity matrices served as targets. The total number of significant connections in the connectograms were compared in HGG, LGG and HC through the two-tailed Student *t*-test (*p* < 0.05). Clinical data regarding language function for LGG and HGG patients were also compared through the two-tailed Student *t*-test (*p* < 0.05). A summary of the study workflow is provided in Supplementary Figure S1. To evaluate any potential confounding effects of tumor size and patients’ age on graph theory results, we conducted a regression and a covariance analysis. Tumor size was evaluated by a neuroradiologist as the product of perpendicular diameters on MRI axial slices. In the case of LGG, the lesion was measured as a hyperintense abnormality on FLAIR images; in HGG, the contrast-enhancing component of the tumor was measured on post-contrast T1-weighted images [19]. 

## 3. Results

### 3.1. Patients

The imaging archive revision identified 245 patients with brain tumors and resting-state data acquired with the same protocol between January 2016 and September 2021. Following our inclusion criteria, 66 patients were excluded due to right-sided tumors, 65 patients were excluded due to prior surgery or treatment, 30 patients were excluded due to pathologic diagnosis other than glioma (21 metastases, 7 lymphoma, 2 radiation necrosis) and 10 patients were excluded due to motion artifacts or the presence of hemorrhagic tumor components, leading to drop-out artifacts on fMRI. Seventy-four patients with gliomas in the left hemisphere (32 LGG; 42 HGG) were selected based on our inclusion criteria. Four patients were excluded due to poor fMRI quality during the data processing phase (interpreted as excessive noise from motion or other artifacts). Ten patients were excluded due to technical failure of image co-registration to the standard space (Figure 1). 

**Figure 2:**
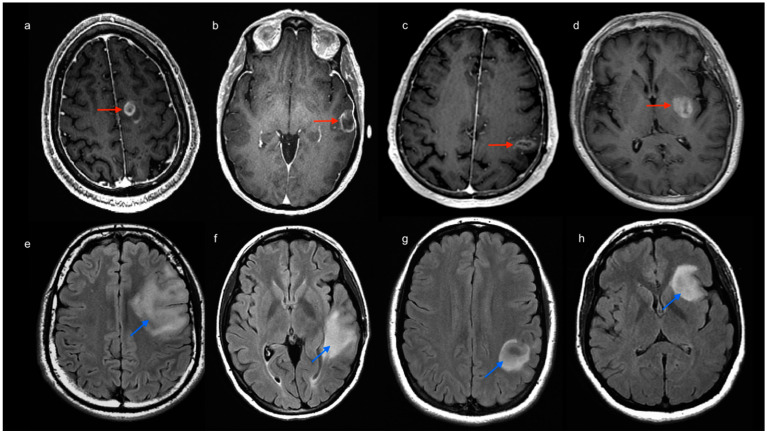
Images above: post-contrast 3D T1-weighted MR scans of four patients with high-grade glioma (image a–d, red arrows). Tumor location was labeled to account for involved lobes as follows: frontal (**a**), temporal (**b**), parietal (**c**) and insular involvement (**d**). Images below: FLAIR-weighted MR scans of four patients with low-grade glioma (image e–h, light blue arrows). Tumor location was labeled to account for involved lobes as follows: frontal (**e**), temporal (**f**), parietal (**g**) and insular involvement (**h**).

Sixty patients with gliomas in the left hemisphere (30 LGG, mean age 40 years, 21 males; 30 HGG, mean age 62 years, 22 males) were therefore recruited for the study. Additionally, 20 HC (mean age 48 years) were included for comparison. Tumor location labeling demonstrated the involvement of the left frontal lobe 20/60 (5 HGG, 15 LGG), left temporal lobe 29/60 (16 HGG, 13 LGG), left parietal lobe 15/60 (11 HGG, 4 LGG) and left insula 17/60 (5 HGG, 12 LGG) (Figure 2). 

No patient showed involvement of the occipital lobe. The Boston Naming Test displayed significantly lower scores in HGG vs. LGG patients (*p* = 0.04). Particularly, HGG patients displayed BNT values between 24 and 60 points (average 46), while LGG values ranged between 51 and 60 points (average 56). The presence of aphasia was different in HGG vs. LGG patients (aphasia in 10/30 HGG vs. 0/30 LGG) in the preoperative setting and after surgery (aphasia in 12/29 HGG vs. 6/27 LGG). Missing datapoints in the presurgical cognitive testing (Boston Naming Test) were related to patient compliance and clinical condition. Missing datapoints in the neurologic/neurosurgical evaluation were related to conservative treatment (no surgery performed) or loss of patient at follow-up. Patient demographics and clinical data regarding language function are available in Supplementary Tables S2 and S3. A statistical description of patients’ age and tumor size is presented in Table S4 and Figure S2. 

### 3.2. Graph Theory Analysis: Whole-Brain and Hemispheric Network Analysis 

**Figure 3:**
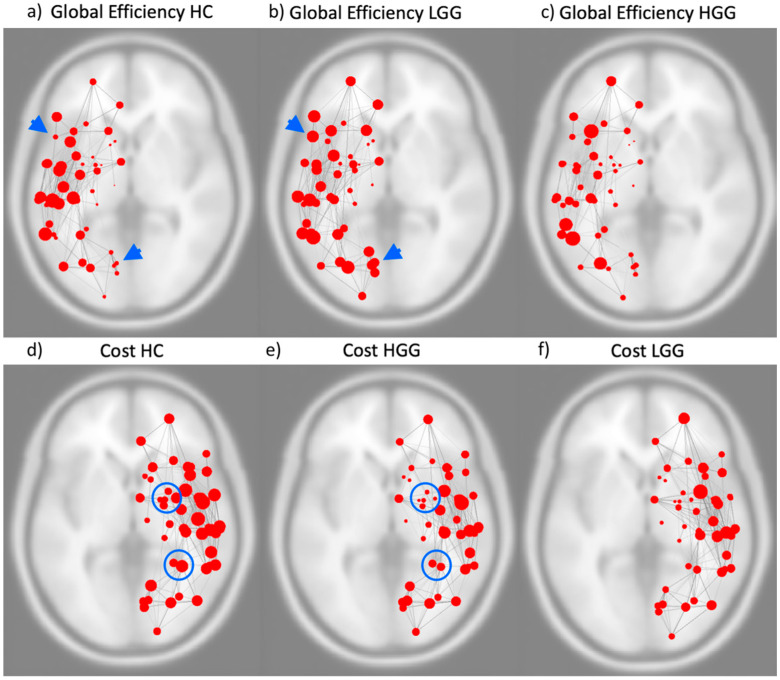
Images above represent the global efficiency of the left hemispheric functional network in healthy controls (**a**), patients with low-grade glioma (LGG) (**b**) and patients with high-grade glioma (HGG) (**c**). Blue arrowheads highlight examples of functional connectivity modification in communities of nodes. Images below represent the cost of the right hemispheric functional network in healthy controls (**d**), patients with HGG (**e**) and patients with LGG (**f**). Significant modifications were detected in the left hemispheric network of LGG compared to HC for global efficiency and in the right hemispheric network of HGG compared to HC for cost. Blue circles highlight examples of significant functional connectivity modifications in communities of nodes. The size of the red nodes represents the value of the respective graph theory metric.

The left hemispheric network was significantly different in LGG compared to HC (increased global efficiency *p* = 0.03). HGG showed significant differences in the right hemispheric network compared to HC (decreased cost *p* = 0.028; decreased degree *p* = 0.028) (Figure 3). 

Further, LGG showed significant differences in left vs. right hemisphere functional network (increased global efficiency *p* = 0.02; decreased local efficiency *p* = 0.01; decreased clustering coefficient *p* = 0.01). No significant differences were found when comparing left and right hemispheric networks in HC or HGG, and no significant differences emerged from the comparison of whole-brain networks. Mean values of the significant graph-theoretical measures for the hemispheric and whole-brain networks are reported in Supplementary Tables S5–S7.

### 3.3. Graph Theory Analysis: Lobar Networks Analysis 

Detailed results for group comparisons according to tumor location are available in Table 1, Table 2 and Table 3. 

All mean values of the significant graph-theoretical measures for the lobar networks are reported in supplementary Tables S8–S14. In brief, frontal tumors showed multiple significant network changes in the left and right hemispheres with both HGG and LGG (*p* < 0.05). Right-sided changes were located in the Superior Frontal Gyrus (SFG), while left-sided changes were localized in the SFG, middle frontal gyrus (MidFG) and Inferior Frontal Gyrus (IFG) Pars Triangularis. 

Temporal tumors showed multiple significant network changes in the left and right hemispheres with both HGG and LGG (*p* < 0.05). Right-sided changes were located in the Superior Temporal Gyrus (STG) posterior division, Middle Temporal Gyrus (MTG) posterior division, MTG temporo-occipital part and Inferior Temporal Gyrus (ITG) anterior division. Left-sided changes were located in STG anterior division, STG posterior division, MTG anterior division and ITG anterior division. 

Parietal and insular tumors showed significant network changes in only the left hemisphere for both LGG and HGG (*p* < 0.05). Particularly, parietal tumors showed network changes in the left Postcentral Gyrus and left Angular Gyrus. Insular LGG were associated with ipsilateral changes, while insular HGG did not produce any significant network changes (Table 3). Table 4 show increased (green) or decreased (red) mean values of significant graph metrics from the lobar analysis. 

No significant difference was found when comparing the total number of significant connections in the connectograms between HGG, LGG and HC. Nevertheless, the total number of connections was higher in LGG than HGG in the majority of the cases (60%), and it was equal in 20%. Only a minority of cases showed higher connections in HGG than LGG (20%). Connectivity diagrams for significant ROIs are reported in Figure 4 and supplementary materials (Supplementary Figures S3–S16).

Regarding potential confounding effects, the linear regression and covariance analyses showed that the correlation and the covariance of both age and tumor size were very low among our dependent and independent variables (Age: Covariance < ±0.2, correlation < ±0.4 and Tumor size: Covariance < ±0.2, correlation < ±0.3). 

## 4. Discussion

We demonstrated that resting-state fMRI and graph-theoretical measures could capture differences in network dynamics associated with tumor grade and location. Graph-theoretical measures demonstrated increased global efficiency for the left hemispheric network in LGG and decreased cost and degree for the right hemispheric network in HGG (Figure 3). We can highlight two main points of clinical significance for our results: (1) the evidence of connectivity disruption in distant areas from HGG supports the notion of the ‘whole-brain’ nature of this disease, differently from LGG, which displayed predominantly local effects within the same hemisphere. (2) By analyzing specific graph-theoretical metrics which describe the efficiency of information transfer in a network, we found that low-grade tumors may show more favorable network changes than high-grade tumors, in agreement with participating patients’ clinical deficits. These findings support the idea that LGG leads to functional reorganization of the eloquent cortex in the same hemisphere resulting in increased efficiency of the network. On the contrary, HGG displayed decreased network connectedness, as well as more profound clinical deficits. 

The increased global efficiency of the left hemispheric network in LGG patients points to higher inter-connectedness of the network compared to HC. Similarly, Derks et al. described increased connectivity between hubs and non-hubs in glioma patients compared to healthy subjects. Such network modifications may be related to intra-hemispheric reorganization, which has been described in LGG, especially in regard to the language network [6,20]. Functional reorganization in LGG has been attributed to their characteristic slow pace of growth, which is believed to facilitate the development of plastic changes. In addition to prior evidence, our results may support the idea of a beneficial intra-hemispheric reorganization in LGG related to increased global efficiency of the left hemispheric network, in agreement with our original hypothesis. Indeed, LGG demonstrated significantly better clinical performance than HGG for language function, which is generally left-lateralized. When comparing the left vs. right hemisphere in LGG, the left side demonstrated increased global efficiency, decreased local efficiency and decreased clustering coefficient. These modifications point to a higher inter-connectedness of the entire left-hemispheric network, with decreased locality (sub-graphs are less inter-connected within their neighborhood and more connected to the entire network). Based on these findings, a type of functional reorganization characterized by the recruitment of additional brain regions within the left hemisphere may be hypothesized, leading to a shift from locality to global efficiency.

In HGG patients, a decrease in all graph-theory metrics compared to HC was seen in the left hemisphere (supplementary Table S6), although without reaching statistical significance. Peritumoral depression of the blood-oxygen level dependent (BOLD) signal is expected in some high-grade tumors due to neurovascular uncoupling [21,22], with possible local effects on connectivity [23]. The fact that left-hemispheric functional changes in HGG did not reach statistical significance in our cohort may be due to relatively small tumor size and/or it may be related to the limitations of our study. In fact, lobar network analysis identified significant left- and right-sided changes in both HGG and LGG. This difference is likely due to the fact that the hemispheric network is averaged across HGG, while the lobar analysis investigates subgroups of HGG according to their lobar location, unraveling smaller effects on connectivity that may be otherwise undetected. 

Hemispheric network analysis in HGG showed significant right-sided changes (decreased cost and degree). Growing evidence supports the idea of gliomas having a global effect on brain functioning, with alterations in brain regions remote from the tumor [12]. HGG is considered a ‘whole-brain’ disease, showing widespread involvement of distant regions even from early stages [24]. The right-hemispheric network in HGG was characterized by decreased cost and degree, which are measures of network centrality expressing the degree of local connectedness of each node (Supplementary Table S1). To a certain extent, these network modifications highlight an effect on brain connectivity which may be characteristic of HGG. Fast-growing tumors may act in a similar way to acute insults such as stroke, leading to the phenomenon of diaschisis. This phenomenon was originally described as a loss of excitability, reduced metabolism and/or blood flow in areas remote to the original lesion [25]. Connectomal diaschisis has been recently described in stroke as changes in the structural and functional connectome, including disconnections between and reorganization of subgraphs, involving areas distant from the original lesion [25]. Our results seem to support a similar effect on functional connectivity driven by the rapid growth of HGG. 

On the other hand, our findings may also point to a certain degree of inter-hemispheric reorganization in HGG. Although plastic changes have been more extensively described in LGG [26], previous studies reported functional reorganization in HGG (e.g., in the cerebral–cerebellar circuits) [27]. Inter-hemispheric language reorganization has been shown in HGG, including translocation of eloquent language areas [28,29,30]. In fact, the median age of glioblastomas at diagnosis is approximately 330 days [31], while brain modifications related to learning new functions can develop within months [32]. This time window may be enough to develop significant network modifications.

**Figure 4:**
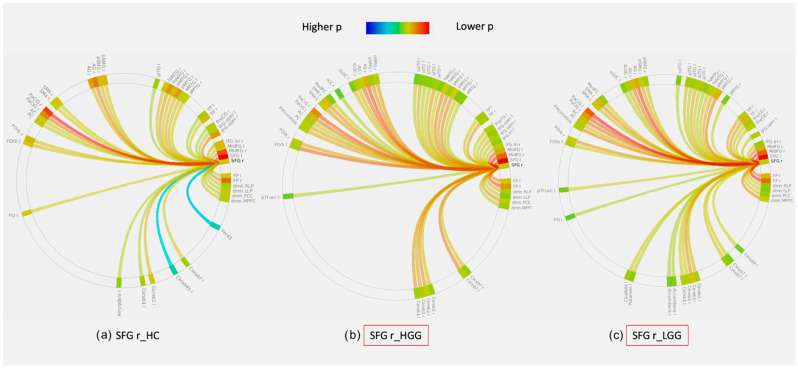
Example of whole-brain connectivity diagrams generated by seeding of the right superior frontal gyrus (SFG r) in healthy controls (HC, (**a**)), left-hemispheric high-grade gliomas (HGG, (**b**)) and left-hemispheric low-grade gliomas (LGG, (**c**)). The red square in diagrams (**b**) and (**c**) indicates that there was a significant difference in functional connectivity in this seed for HGG and LGG compared to HC (**a**). The color scale of the links represents their statistical significance (*p*-value FDR corrected, only links with *p* < 0.05 are included). Regions of interest (ROI) in the diagrams are labeled as per the AAL atlas (available in CONN toolbox at https://web.conn-toolbox.org, accessed on 3 March 2022).

The effect of tumor location on brain plasticity is rarely investigated. From our dedicated analysis, tumor location appears to be crucial for network changes. While frontal and temporal tumors showed bilateral functional modifications, parietal and insular tumors appeared to display only local effects (i.e., functional changes in the left hemisphere). Notably, essential functions such as language are primarily lateralized to the left frontal and temporal lobes [33], while the parietal and insular lobes host secondary language areas [34]. Nenning et al. showed that unilateral tumors are associated with inter-hemispheric reorganization and that the proximity of tumor location is more linked to distributed network deterioration than anatomical distance [35]. Eloquent regions may act as connectors in the functional network [8], whose damage leads to deeper and wider modifications of the network organization than peripheral areas, as supported by neuro-computational models [36]. Interestingly, while frontal tumors showed mixed connectivity changes, temporal tumors led to a decrease in all graph metrics in both LGG and HGG of both hemispheres (Table 4). Briganti et al. reported a stronger decrease in left hemispheric connectivity in posterior vs. anterior tumors by studying the language network with a pseudo-resting state [37]. These results may suggest that lesions in the left temporal lobe have a more detrimental effect on connectivity than other locations and that beneficial plastic changes may be more likely to occur with a frontal than a temporal tumor. The presence of a tumor in the left frontal lobe was associated with significant modification of connectivity at the level of the right SFG in both LGG and HGG patients vs. HC (Figure 4). However, while the node had increased global efficiency, local efficiency, degree and decreased average path length in LGG vs. HC, the opposite was true for HGG vs. HC. As a consequence, the node was more integrated into LGG than HGG. This finding may support the idea that such right-sided network modification was more favorable in LGG than in HGG. Despite this promising finding, connectivity changes in other nodes were less indicative of a specific pattern (Table 4).

The evaluation of whole-brain connectograms, obtained from seeding significant nodes in the lobar analysis, revealed no single pattern for all patients (Figure 4 and Supplementary Figures S3–S16). However, in the majority of cases (80%), the same nodes in HGG showed an equal (20%) or lower (60%) number of significant connections (*p* < 0.05, FDR corrected) when compared to LGG. Global attenuation of the resting-state fMRI signal induced by brain tumors was previously related to remodeling of the neurovasculature [38] and may also reflect the connectivity impairment caused by tumor invasion. This may partially justify the lower clinical performance of HGG patients.

This study has some limitations. First, the number of patients was somehow small due to our inclusion criteria, which required presurgical LGG patients with comparable resting-state data and the absence of motion and susceptibility artifacts. We also excluded patients with prior chemotherapy or radiation, further reducing our sample size. We made this choice to avoid connectivity modifications caused by the treatment, as documented in prior studies [39,40], which would have acted as a confounding factor in our analysis. LGG patients were younger than HGG patients, as expected from different age-specific incidences. Patient groups according to the location labels were variably unbalanced, limiting the generalizability of our results. Comparison of graph-theoretical metrics in tumors involving specific eloquent areas was not performed due to the limited number of subjects. There was a considerable amount of missing BNT data due to patient compliance/clinical state at the moment of the examination. Therefore, correlations between BNT scores and specific graph-theory metrics were not performed. 

As a final consideration, many variables can potentially affect functional connectivity in the setting of brain tumors. Tumor growth, local aggressivity and invasion likely represent drivers of plastic changes [6] and are affected by genetic mutations, as well as epigenetic alterations. The effect of treatment on connectivity has been clearly demonstrated by previous studies [39,41]. Although not emerging from our results, the patient’s age may affect functional changes since brain ageing is related to diminished plastic potential [42]. The patient’s sex may also have an important effect due to well-known differences in connectivity patterns [43]. Tumor size differences may also affect brain connectivity. Future studies are called to confirm our findings in the larger population, to further explore the effects of other patient- and tumor-related variables on brain connectivity, as well as the clinical meaning of functional changes. 

## 5. Conclusions

We demonstrated that tumors of different grades have different effects on functional connectivity. While LGG leads to increased efficiency of the surrounding functional network, HGG seems to disrupt brain connectivity in remote areas, recalling the phenomenon of diaschisis. Tumor location appears to influence the pattern of reorganization, including recruitment of the contralateral hemisphere. LGG may show more favorable connectivity changes than HGG. Nevertheless, the effect of brain tumors on connectivity remains complex, with different coexistent network modifications that cannot be ascribed to a single pattern. As a consequence, the compensatory nature and clinical meaning of functional changes remain to be fully understood, and no definite conclusions can be drawn at the current stage. If confirmed by future studies, the ability to discriminate between ‘maladaptive’ (detrimental, as seen in HGG) and ‘adaptive’ (beneficial, as seen in LGG) functional reorganization based on graph theory metrics may provide biomarkers to select patients for surgery and monitor recovery. 

## Figures and Tables

**Table 1:** Graph-theory statistics results for gliomas involving the frontal lobe. Significance is reported as FDR corrected *p*-value.

Frontal Tumors	HGG/Controls	*p*-Value	LGG/Controls	*p*-Value	HGG/LGG	*p*-Value
Global Efficiency			SFG r	0.006		
Local Efficiency	SFG r	0.045			SFG r	0.045
Betweenness Centrality	IFG tri l	0.006	SFG r	0.025	IFG tri l	0.001
			SFG l	0.012		
Cost			SFG r	0.028		
Average Path Length			SFG r	0.003	SFG r	0.045
			SFG l	0.043		
Clustering Coefficient	SFG r	0.017	MidFG l	0.042		
	IFG tri l	0.016				
Degree			SFG r	0.028		

HGG = high-grade gliomas; LGG = low-grade gliomas; MidFG l = middle frontal gyrus left; SFG l = superior frontal gyrus left; SFG r = superior frontal gyrus right; IFG tri l = left inferior frontal gyrus pars triangularis.

**Table 2:** Graph-theory statistics results for gliomas involving the temporal lobe. Significance is reported as FDR corrected *p*-value.

Temporal Tumors	HGG/Controls	*p*-Value	LGG/Controls	*p*-Value	HGG/LGG	*p*-Value
Global Efficiency	pSTG l	0.010	aSTG l	0.027		
Local Efficiency	pMTG r	0.044	pSTG l	0.003		
Betweenness Centrality	pSTG r	0.040	aMTG l	0.030		
Cost	pMTG r	0.022	aSTG l	0.018	pMTG r	0.031
	toMTG r	0.032	pSTG l	0.008		
	pSTG l	0.032	aMTG l	0.017		
			aITG l	0.038		
Clustering Coefficient			aITG r	0.017		
			pSTG l	0.018		
Degree	pMTG r	0.022	aSTG l	0.018	pMTG r	0.031
	toMTG r	0.032	pSTG l	0.008		
	pSTG l	0.032	aMTG l	0.017		
			aITG l	0.038		

HGG = high-grade gliomas; ITG = inferior temporal gyrus (l = left; r = right; a = anterior division); LGG = low-grade gliomas; MTG = middle temporal gyrus (l = left; r = right; a = anterior division; p = posterior division; to = temporo-occipital); STG = superior temporal gyrus (l = left; r = right; a = anterior division; p = posterior division).

**Table 3:** Graph-theory statistics results for gliomas involving the parietal lobe and insula. Significance is reported as FDR corrected *p*-value.

**Parietal Tumors**	**HGG/Controls**	***p*** **-Value**	**LGG/Controls**	***p*** **-Value**
Global Efficiency	AG l	0.0434		
Local Efficiency	PostCG l	0.0145		
Betweenness Centrality	PostCG l	0.0051	PostCG l	0.0236
Average Path Length	AG l	0.0394		
**Insular Tumors**	**HGG/Controls**	***p*** **-Value**	**LGG/Controls**	***p*** **-Value**
Cost			IC l	0.0161
Degree			IC l	0.0161

AG l = angular gyrus left; HGG = high-grade gliomas; IC l = insular cortex left; LGG = low-grade gliomas; PostCG l = post-central gyrus left.

**Table 4:** Increased (green) or decreased (red) mean values of significant graph metrics.

SFG r	HGG/HC	LGG/HC	pSTG r	HGG/HC	
	Local Efficiency	Global Efficiency		Betweenness	
	Clustering Coefficient	Betweenness	pSTG l	HGG/HC	LGG/HC
		Cost		Global Efficiency	Local Efficiency
		Average Path Length		Cost	Cost
		Degree		Degree	Clustering Coefficient
SFG l		LGG/HC			Degree
		Average Path Length	aSTG l		LGG/HC
MidFG l		LGG/HC			Global Efficiency
		Clustering Coefficient			Cost
IFG tri l	HGG/HC				Degree
	Betweenness		aITG l		LGG/HC
	Clustering Coefficient				Cost
pMTG r	HGG/HC				Degree
	Local Efficiency		aITG r		LGG/HC
	Cost				Clustering Coefficient
	Degree		AG l	HGG/HC	
aMTG l		LGG/HC		Global Efficiency	
		Betweenness		Average Path Length	
		Cost	PostCG l	HGG/HC	LGG/HC
		Degree		Local Efficiency	Betweenness
				Betweenness	
toMTG r	HGG/HC		IC l		LGG/HC
	Cost				Cost
	Degree				Degree

AG l = angular gyrus left; HGG = high-grade gliomas; IC l = insular cortex left; IFG tri l = left inferior frontal gyrus pars triangularis; ITG = inferior temporal gyrus (l = left; r = right; a = anterior division); LGG = low-grade gliomas; MidFG l = middle frontal gyrus left; MTG = middle temporal gyrus (l = left; r = right; a = anterior division; p = posterior division; to = temporo-occipital); PostCG l = post-central gyrus left; STG = superior temporal gyrus (l = left; r = right; a = anterior division; p = posterior division); SFG = superior frontal gyrus (l = left; r = right).

## Data Availability

Anonymized patient data is available upon reasonable request to the authors.

## Supplementary Materials

The following supporting information can be downloaded at: https://www.mdpi.com/article/10.3390/cancers14143327/s1, Figure S1: Summary of the study population and workflow; Figure S2: Distribution of tumor size and patients’ age for HGG and LGG in our study; Figure S3: Whole-brain connectivity diagrams of left superior frontal gyrus; Figure S4: Whole-brain connectivity diagrams of the left middle frontal gyrus; Figure S5: Whole-brain connectivity diagrams of the left inferior frontal gyrus, pars triangularis; Figure S6: Whole-brain connectivity diagrams of the right superior temporal gyrus, posterior division; Figure S7: Whole-brain connectivity diagrams of the left superior temporal gyrus, posterior division; Figure S8: Whole-brain connectivity diagrams of the left inferior temporal gyrus, anterior division; Figure S9: Whole-brain connectivity diagrams of the right inferior temporal gyrus, anterior division; Figure S10: Whole-brain connectivity diagrams of the left middle temporal gyrus, anterior division; Figure S11: Whole-brain connectivity diagrams of the left superior temporal gyrus, anterior division; Figure S12: Whole-brain connectivity diagrams of the right middle temporal gyrus, temporo-occipital division; Figure S13: Whole-brain connectivity diagrams of the right middle temporal gyrus posterior division; Figure S14: Whole-brain connectivity diagrams of the left post-central gyrus; Figure S15: Whole-brain connectivity diagrams of the left angular gyrus; Figure S16: Whole-brain connectivity diagrams of the left insular cortex; Table S1: Summary of graph-theoretical metrics investigated in this study (available at https://web.conn-toolbox.org, accessed on 3 March 2022); Table S2: Clinical and demographic data for HGG patients; Table S3: Clinical and demographic data for LGG patients; Table S4: Statistical description of tumor size and patients’ age for HGG and LGG in our study; Table S5: Whole brain network mean values of graph-theory results; Table S6: Left hemispheric network mean values of graph-theory results; Table S7: Right hemispheric network mean values of graph-theory results; Table S8: Mean values of significant graph-theory results for gliomas involving the frontal lobe; Table S9: Mean values of significant graph-theory results for gliomas involving the frontal lobe; Table S10: Mean values of significant graph-theory results for gliomas involving the temporal lobe; Table S11: Mean values of significant graph-theory results for gliomas involving the temporal lobe; Table S12: Mean values of significant graph-theory results for gliomas involving the temporal lobe; Table S13: Mean values of significant graph-theory results for parietal gliomas; Table S14: Mean values of significant graph-theory results for insular gliomas.
