# Supplementary material for: Brain Functional Connectivity in Low- and High-Grade Gliomas: Differences in Network Dynamics Associated with Tumor Grade and Location
Source: Cancers (Basel). 2022 Jul 8;14(14):3327. doi: 10.3390/cancers14143327 (PMC9324249; doi:10.3390/cancers14143327)
Supplement: Supplementary file 1 [file cancers-14-03327-s001.zip › cancers-1761870-supplementary.pdf]

SUPPLEMENTARY MATERIAL

Figure S1. Summary of the study population and workflow

- Study population: **30** low-grade (LGG), **30** high-grade (HGG) **left-hemispheric glioma** patients and **20** healthy controls (HC)

30 LGG
mean age 40 years,
21 males

30 HGG
mean age 61 years,
22 males

20 HC
mean age 48 years,
12 males

- Since the tumors were left-hemispheric, we investigated patients' language performance as example of left-lateralized function through the Boston Naming Test, to identify possible clinical correlates of network changes

- We applied graph-theory on resting-state fMRI data in three separate analyses: 1) whole-brain functional networks; 2) hemispheric networks; 3) lobar networks in sub-groups of patients divided by tumor location

1)

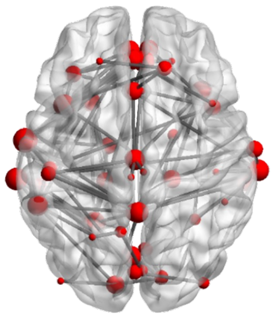

2)

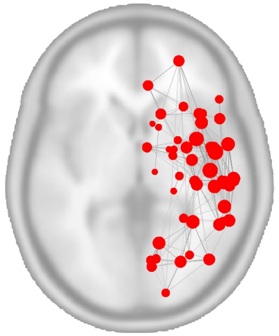

3)

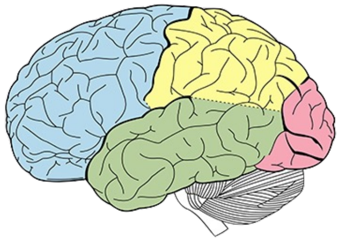

- Seven graph-theoretical metrics were calculated in every functional network (FDR corrected, $p<0.05$). Two-tailed Student t-test or Mann-Whitney U-test ($p<0.05$) were used to compare the results in **LGG vs. HC** and **HGG vs. HC**.

Figure S2. Distribution of tumor size and patients' age in our study population of HGG and LGG

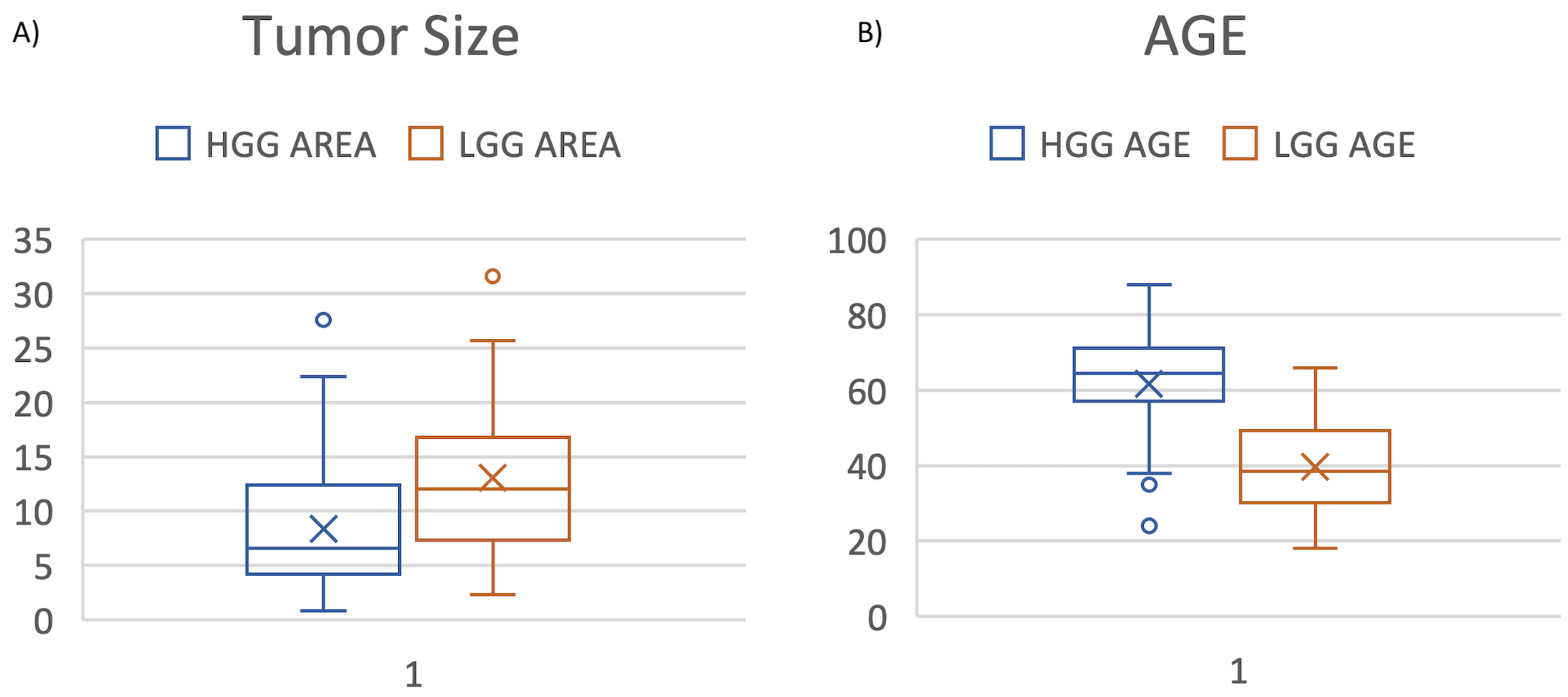

Box-plots representing the distribution of tumor size (A) and patients' age (B) in HGG (blue) and LGG (orange). Tumor size is expressed in cm^{2}, patients' age is expressed in years

Figure S3. Whole-brain connectivity diagrams of left superior frontal gyrus

Higher p — Lower p

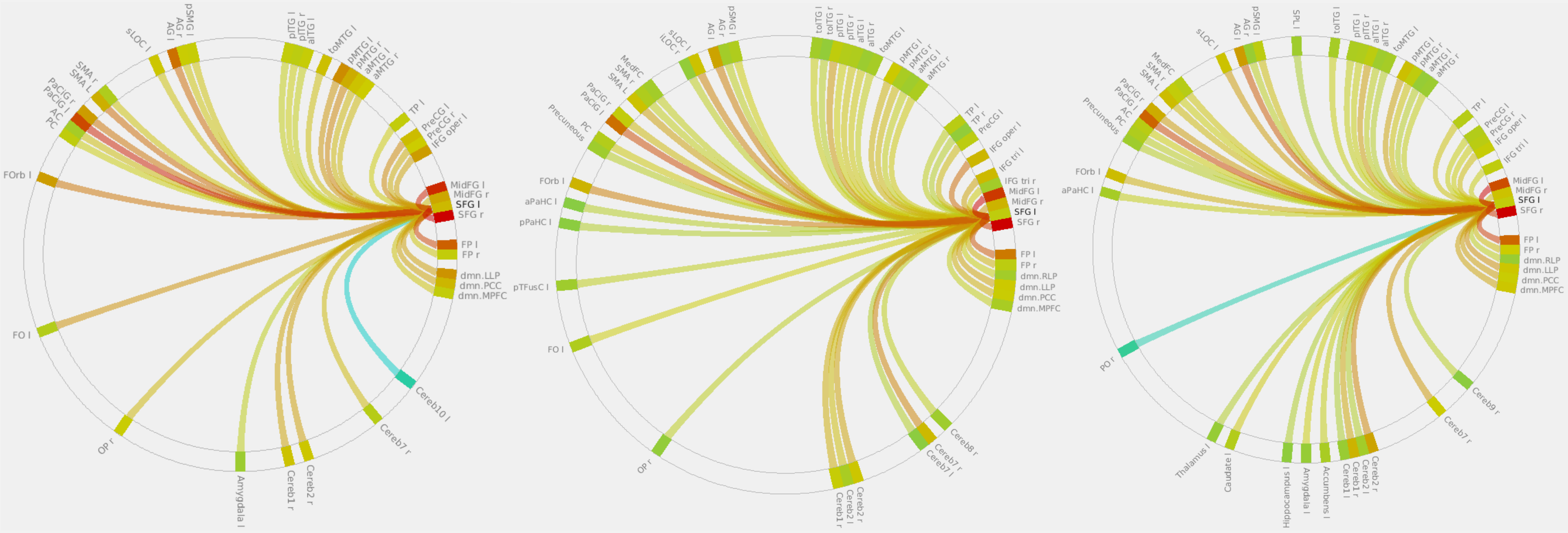

a) SFG l_HC

b) SFG l_HGG

c) SFG l_LGG

Whole-brain connectivity diagrams generated by seeding the left superior frontal gyrus (SFG l) in healthy controls (HC), left-hemispheric high-grade gliomas (HGG) and left-hemispheric low-grade gliomas (LGG). The red square in diagram c) indicates that there was a significant difference in functional connectivity in this seed for LGG compared to HC. The color scale of the links represents their statistical significance (p-value FDR corrected, only links with $p<0.05$ are included). Regions of interest (ROI) in the diagrams are labeled as per the AAL atlas (available in CONN toolbox at https://web.conn-toolbox.org)

Figure S4. Whole-brain connectivity diagrams of the left middle frontal gyrus

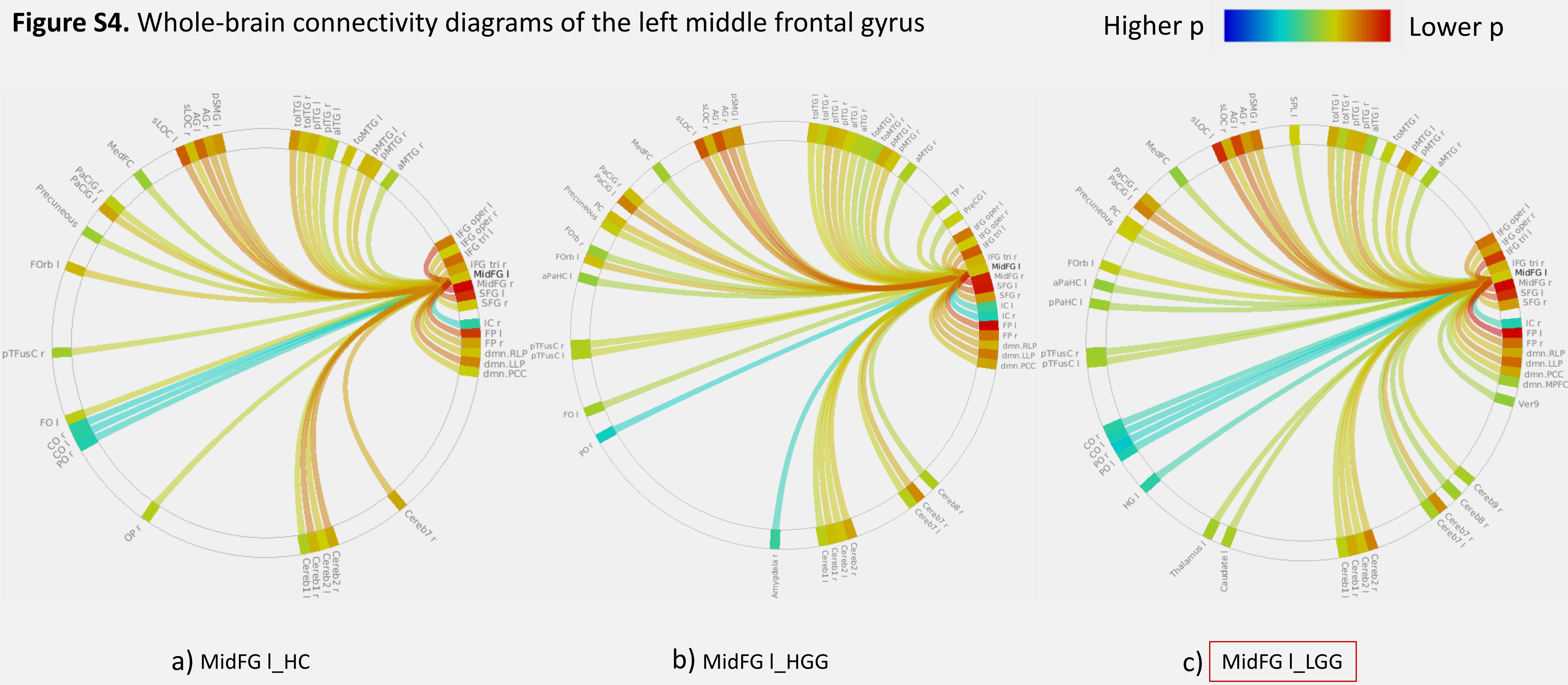

Whole-brain connectivity diagrams generated by seeding the left middle frontal gyrus (MidFG l) in healthy controls (HC), left-hemispheric high-grade gliomas (HGG) and left-hemispheric low-grade gliomas (LGG). The red square in diagram c) indicates that there was a significant difference in functional connectivity in this seed for LGG compared to HC. The color scale of the links represents their statistical significance (p-value FDR corrected, only links with $p<0.05$ are included). Regions of interest (ROI) in the diagrams are labeled as per the AAL atlas (available in CONN toolbox at https://web.conn-toolbox.org)

Figure S5. Whole-brain connectivity diagrams of the left inferior frontal gyrus, pars triangularis

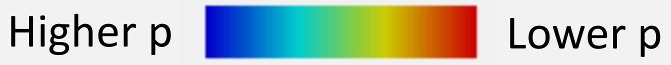

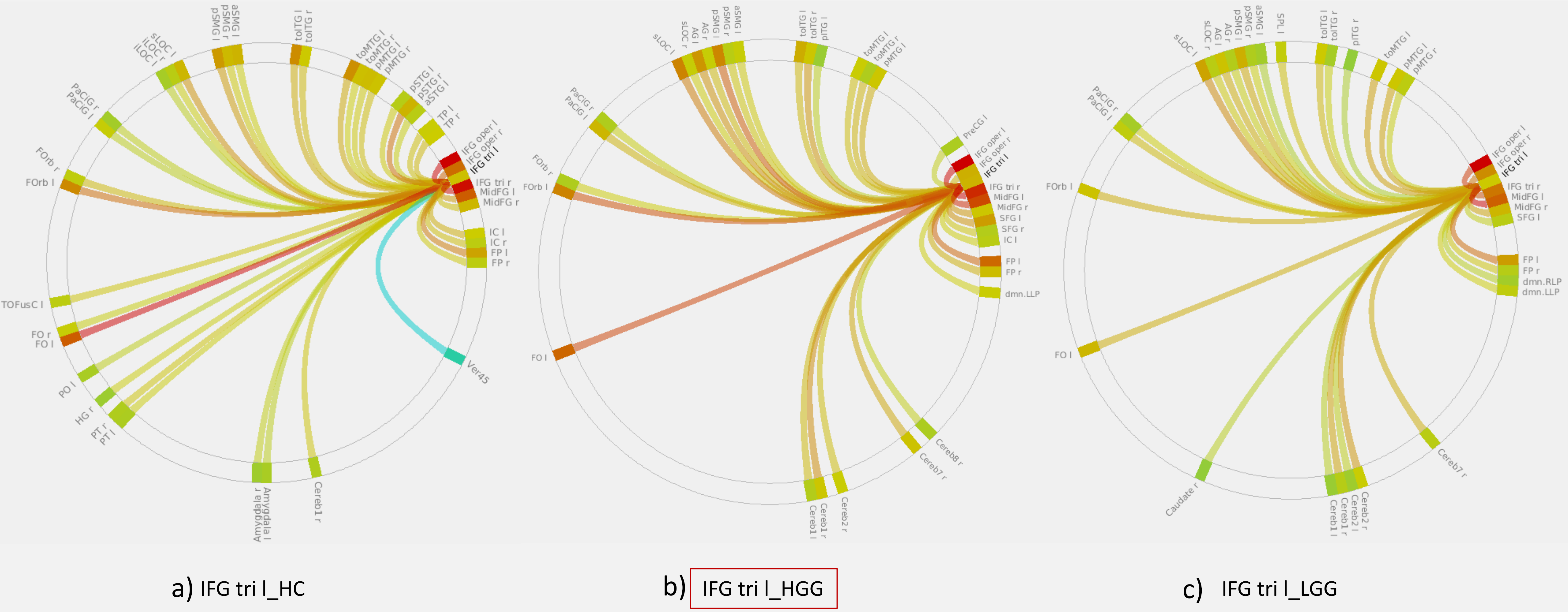

Whole-brain connectivity diagrams generated by seeding the pars triangularis of the left inferior frontal gyrus (IFG tri l) in healthy controls (HC), left-hemispheric high-grade gliomas (HGG) and left-hemispheric low-grade gliomas (LGG). The red square in diagram b) indicates that there was a significant difference in functional connectivity in this seed for HGG compared to HC. The color scale of the links represents their statistical significance (p-value FDR corrected, only links with $p<0.05$ are included). Regions of interest (ROI) in the diagrams are labeled as per the AAL atlas (available in CONN toolbox at https://web.conn-toolbox.org)

Figure S6. Whole-brain connectivity diagrams of the right superior temporal gyrus, posterior division

Higher p Lower p

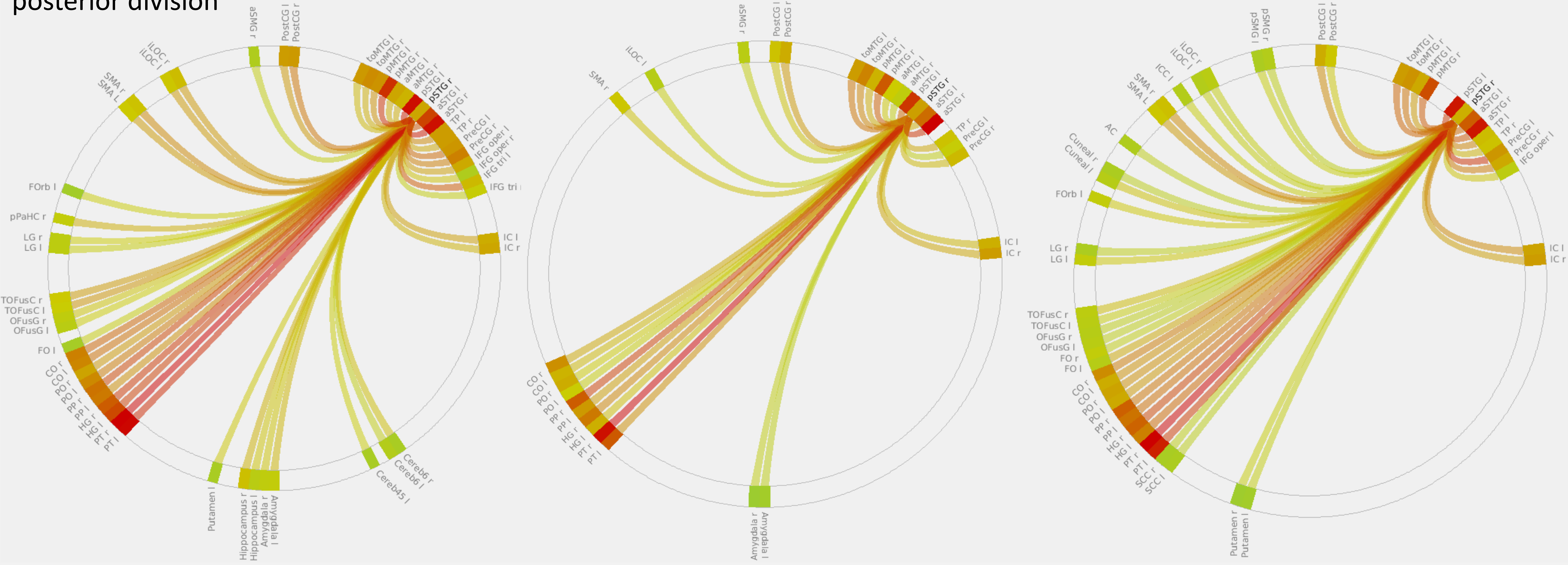

a) pSTGr_HC

b) pSTGr_HGG

c) pSTGr_LGG

Whole-brain connectivity diagrams generated by seeding the posterior division of the right superior temporal gyrus (pSTGr) in healthy controls (HC), left-hemispheric high-grade gliomas (HGG) and left-hemispheric low-grade gliomas (LGG). The red square in diagram b) indicates that there was a significant difference in functional connectivity in this seed for HGG compared to HC. The color scale of the links represents their statistical significance (p-value FDR corrected, only links with p<0.05 are included). Regions of interest (ROI) in the diagrams are labeled as per the AAL atlas (available in CONN toolbox at https://web.conn-toolbox.org)

Figure S7. Whole-brain connectivity diagrams of the left superior temporal gyrus, posterior division

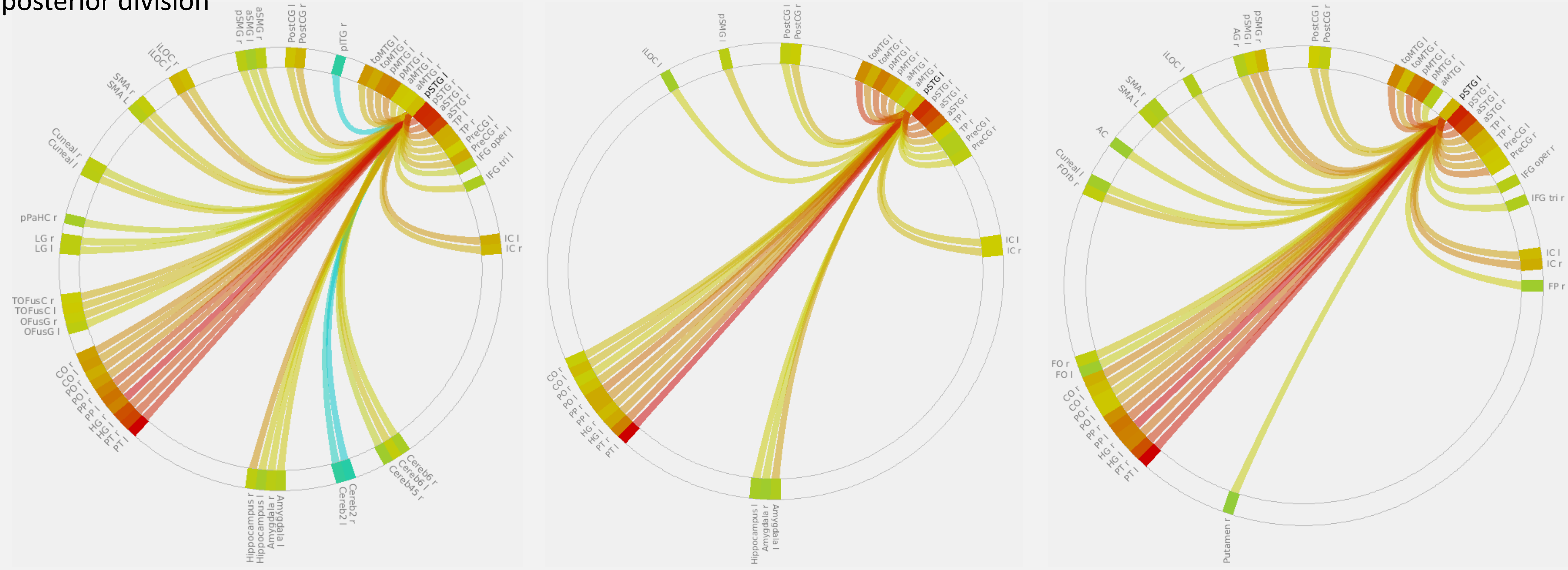

a) pSTG l_HC

b) pSTG l_HGG

c) pSTG l_LGG

Whole-brain connectivity diagrams generated by seeding the posterior division of the left superior temporal gyrus (pSTG l) in healthy controls (HC), left-hemispheric high-grade gliomas (HGG) and left-hemispheric low-grade gliomas (LGG). The red square in diagram b) and c) indicates that there was a significant difference in functional connectivity in this seed for HGG and LGG compared to HC. The color scale of the links represents their statistical significance (p-value FDR corrected, only links with $p<0.05$ are included). Regions of interest (ROI) in the diagrams are labeled as per the AAL atlas (available in CONN toolbox at https://web.conn-toolbox.org)

Figure S8. Whole-brain connectivity diagrams of the left inferior temporal gyrus, anterior division

Higher p — Lower p

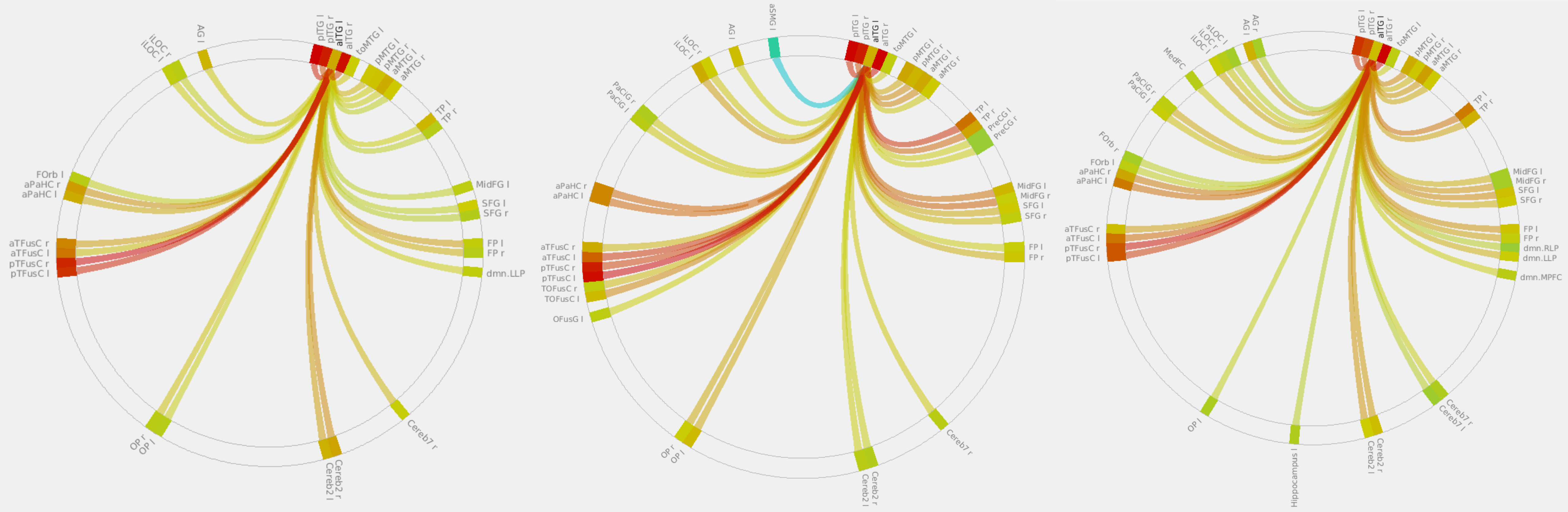

a) aITG l_HC

b) aITG l_HGG

c) aITG l_LGG

Whole-brain connectivity diagrams generated by seeding the anterior division of the left inferior temporal gyrus (aITG l) in healthy controls (HC), left-hemispheric high-grade gliomas (HGG) and left-hemispheric low-grade gliomas (LGG). The red square in diagram c) indicates that there was a significant difference in functional connectivity in this seed for LGG compared to HC. The color scale of the links represents their statistical significance (p-value FDR corrected, only links with $p<0.05$ are included). Regions of interest (ROI) in the diagrams are labeled as per the AAL atlas (available in CONN toolbox at https://web.conn-toolbox.org)

Figure S9. Whole-brain connectivity diagrams of the right inferior temporal gyrus, anterior division

Higher p — Lower p

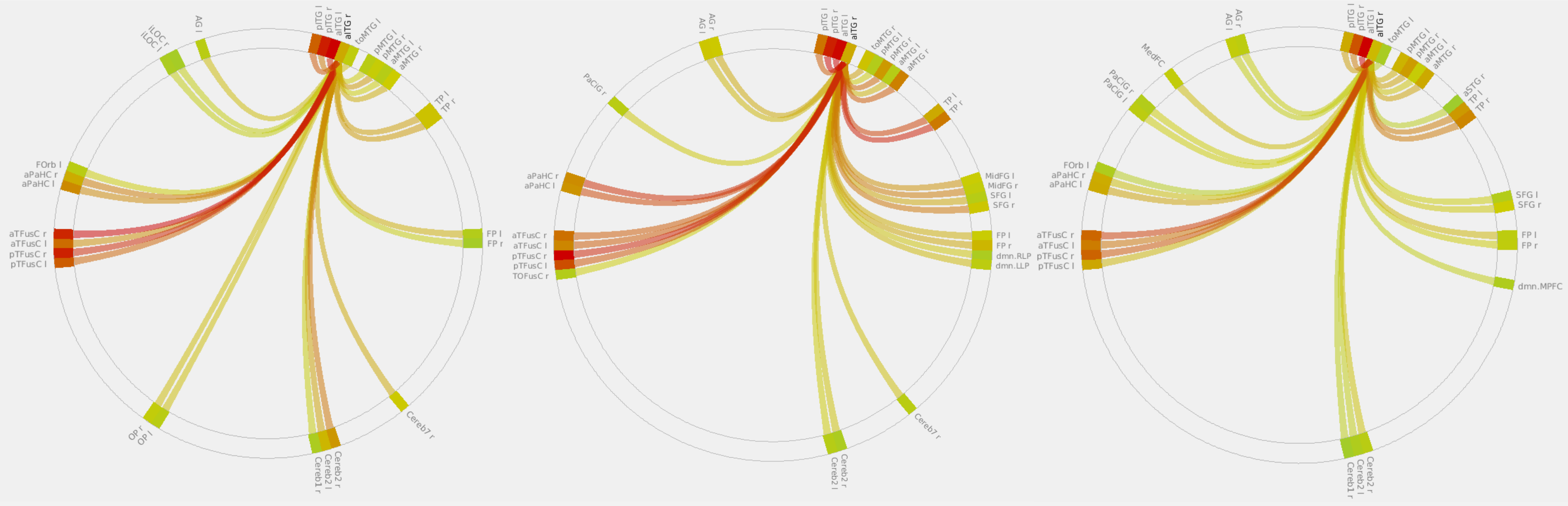

a) aITG r_HC

b) aITG r_HGG

c) aITG r_LGG

Whole-brain connectivity diagrams generated by seeding the anterior division of the right inferior temporal gyrus (aITG r) in healthy controls (HC), left-hemispheric high-grade gliomas (HGG) and left-hemispheric low-grade gliomas (LGG). The red square in diagram c) indicates that there was a significant difference in functional connectivity in this seed for LGG compared to HC. The color scale of the links represents their statistical significance (p-value FDR corrected, only links with $p<0.05$ are included). Regions of interest (ROI) in the diagrams are labeled as per the AAL atlas (available in CONN toolbox at https://web.conn-toolbox.org)

Figure S10. Whole-brain connectivity diagrams of the left middle temporal gyrus, anterior division

Higher p — Lower p

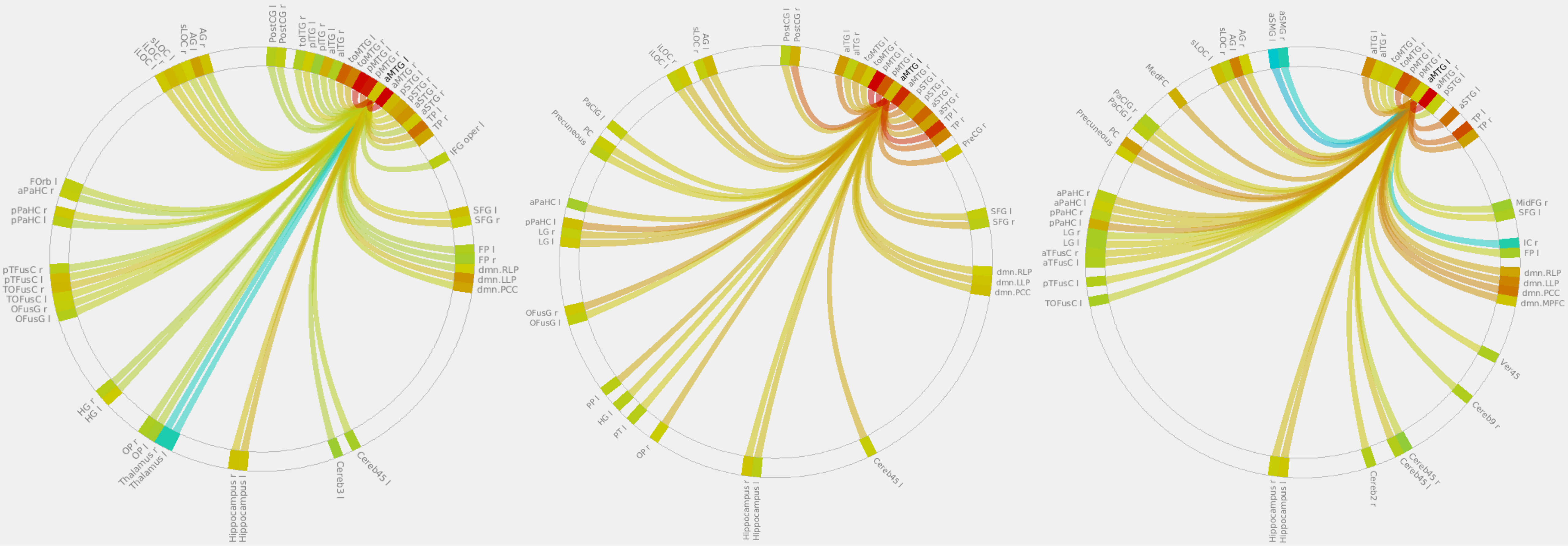

a) aMTG l_HC

b) aMTG l_HGG

c) aMTG l_LGG

Whole-brain connectivity diagrams generated by seeding the anterior division of the left middle temporal gyrus (aMTG l) in healthy controls (HC), left-hemispheric high-grade gliomas (HGG) and left-hemispheric low-grade gliomas (LGG). The red square in diagram c) indicates that there was a significant difference in functional connectivity in this seed for LGG compared to HC. The color scale of the links represents their statistical significance (p-value FDR corrected, only links with p<0.05 are included). Regions of interest (ROI) in the diagrams are labeled as per the AAL atlas (available in CONN toolbox at https://web.conn-toolbox.org)

Figure S11. Whole-brain connectivity diagrams of the left superior temporal gyrus, anterior division

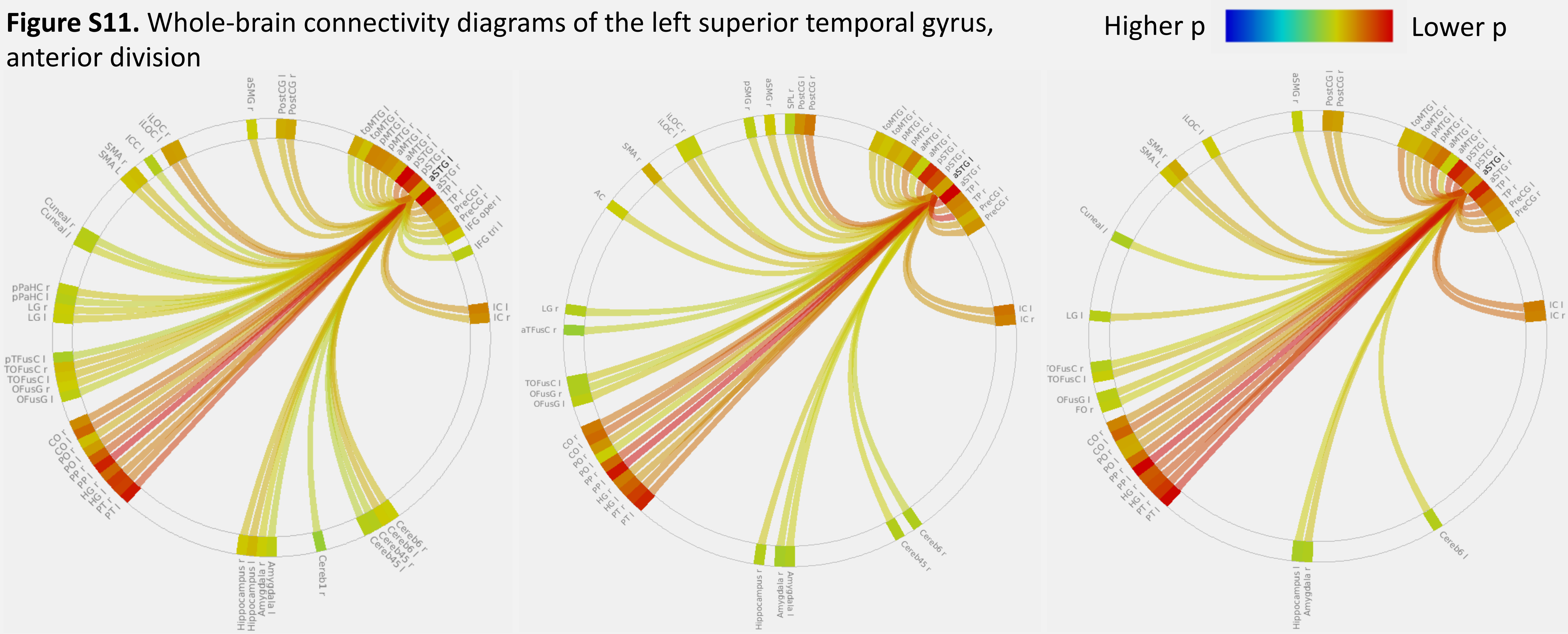

a) aSTG l_HC b) aSTG l_HGG c) aSTG l_LGG

Whole-brain connectivity diagrams generated by seeding the anterior division of the left superior temporal gyrus (aSTG l) in healthy controls (HC), left-hemispheric high-grade gliomas (HGG) and left-hemispheric low-grade gliomas (LGG). The red square in diagram c) indicates that there was a significant difference in functional connectivity in this seed for LGG compared to HC. The color scale of the links represents their statistical significance (p-value FDR corrected, only links with $p<0.05$ are included). Regions of interest (ROI) in the diagrams are labeled as per the AAL atlas (available in CONN toolbox at https://web.conn-toolbox.org)

Figure S12. Whole-brain connectivity diagrams of the right middle temporal gyrus, temporo-occipital division

Higher p 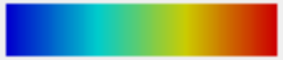 Lower p

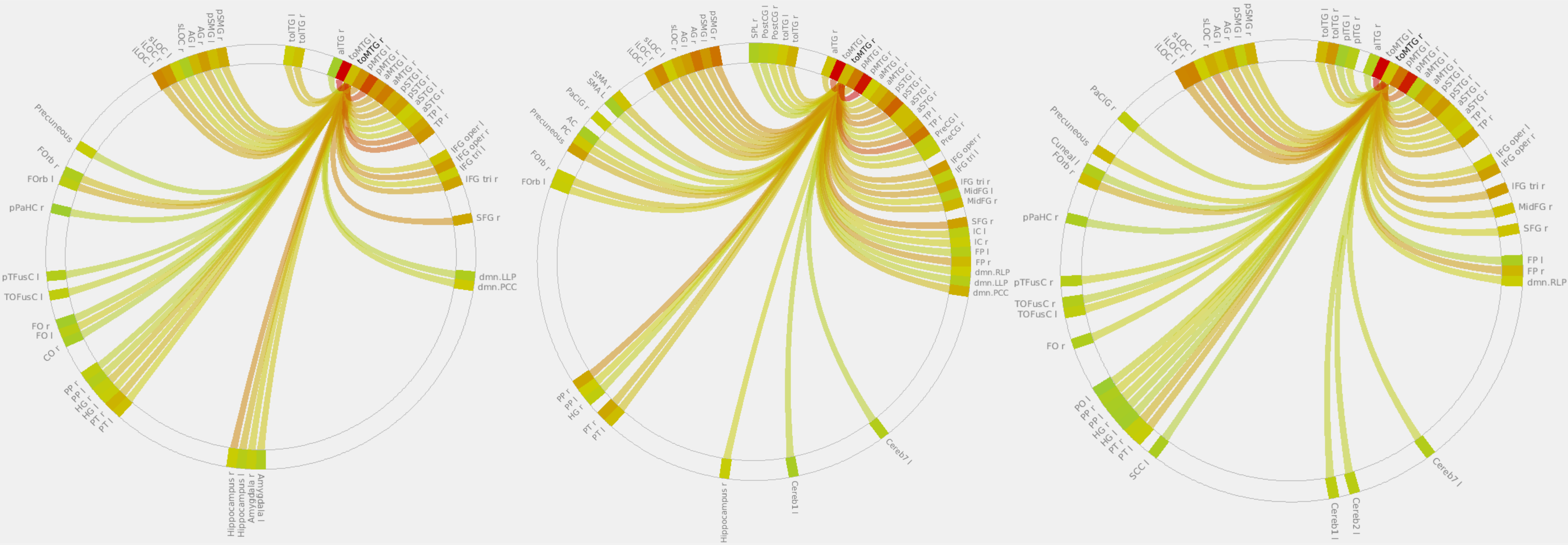

a) toMTG r_HC

b) toMTG r_HGG

c) toMTG r_LGG

Whole-brain connectivity diagrams generated by seeding the temporo-occipital division of the right middle temporal gyrus (toMTG r) in healthy controls (HC), left-hemispheric high-grade gliomas (HGG) and left-hemispheric low-grade gliomas (LGG). The red square in diagram b) indicates that there was a significant difference in functional connectivity in this seed for HGG compared to HC. The color scale of the links represents their statistical significance (p-value FDR corrected, only links with p<0.05 are included). Regions of interest (ROI) in the diagrams are labeled as per the AAL atlas (available in CONN toolbox at https://web.conn-toolbox.org)

Figure S13. Whole-brain connectivity diagrams of the right middle temporal gyrus posterior division

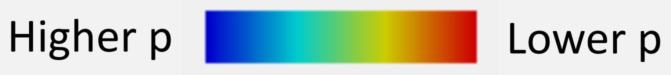

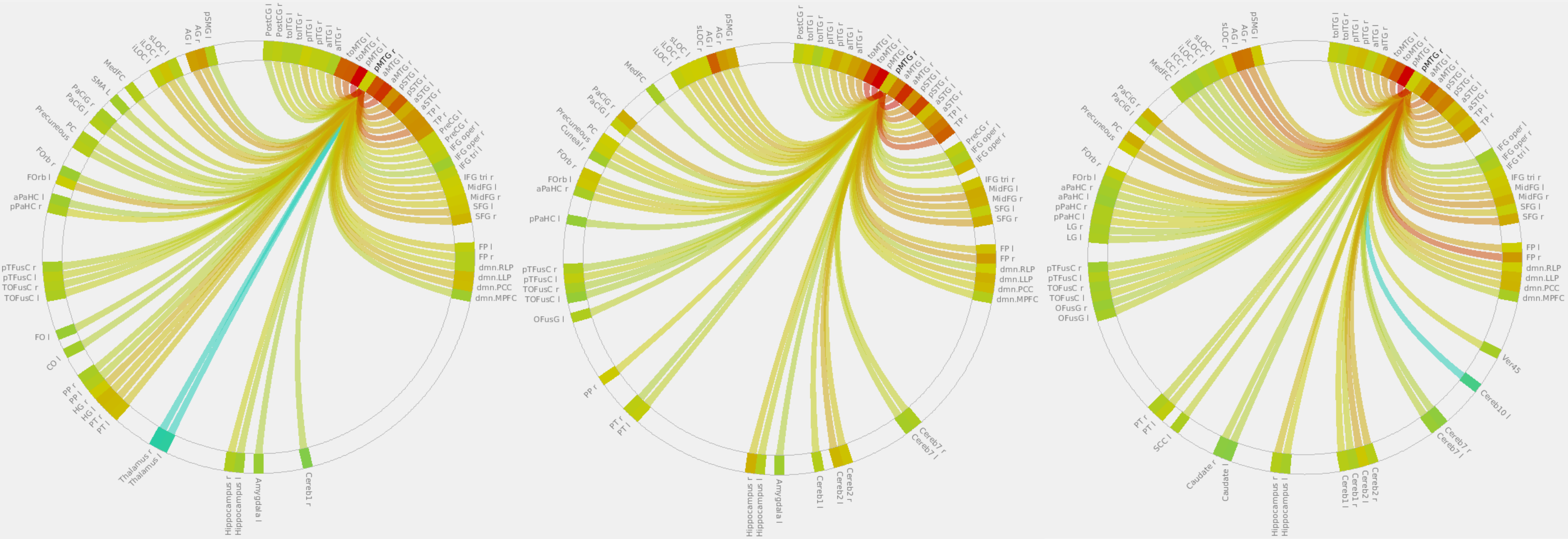

a) pMTG r_HC

b) pMTG r_HGG

c) pMTG r_LGG

Whole-brain connectivity diagrams generated by seeding the posterior division of the right middle temporal gyrus (pMTG r) in healthy controls (HC), left-hemispheric high-grade gliomas (HGG) and left-hemispheric low-grade gliomas (LGG). The red square in diagram b) indicates that there was a significant difference in functional connectivity in this seed for HGG compared to HC. The color scale of the links represents their statistical significance (p-value FDR corrected, only links with $p<0.05$ are included). Regions of interest (ROI) in the diagrams are labeled as per the AAL atlas (available in CONN toolbox at https://web.conn-toolbox.org)

Figure S14. Whole-brain connectivity diagrams of the left post-central gyrus

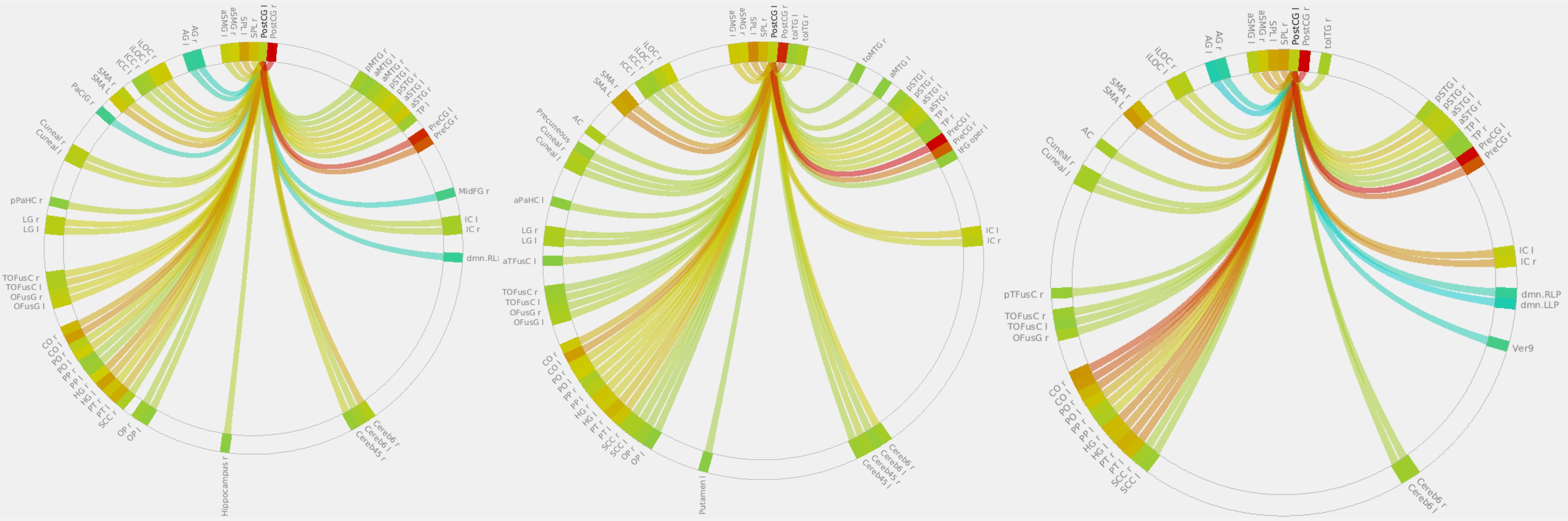

a) PostCG l_HC

b) PostCG l_HGG

c) PostCG l_LGG

Whole-brain connectivity diagrams generated by seeding the left post-central gyrus (PostCG l) in healthy controls (HC), left-hemispheric high-grade gliomas (HGG) and left-hemispheric low-grade gliomas (LGG). The red square in diagram b) and c) indicates that there was a significant difference in functional connectivity in this seed for HGG and LGG compared to HC. The color scale of the links represents their statistical significance (p-value FDR corrected, only links with $p<0.05$ are included). Regions of interest (ROI) in the diagrams are labeled as per the AAL atlas (available in CONN toolbox at https://web.conn-toolbox.org)

Figure S15. Whole-brain connectivity diagrams of the left angular gyrus

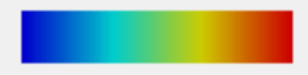

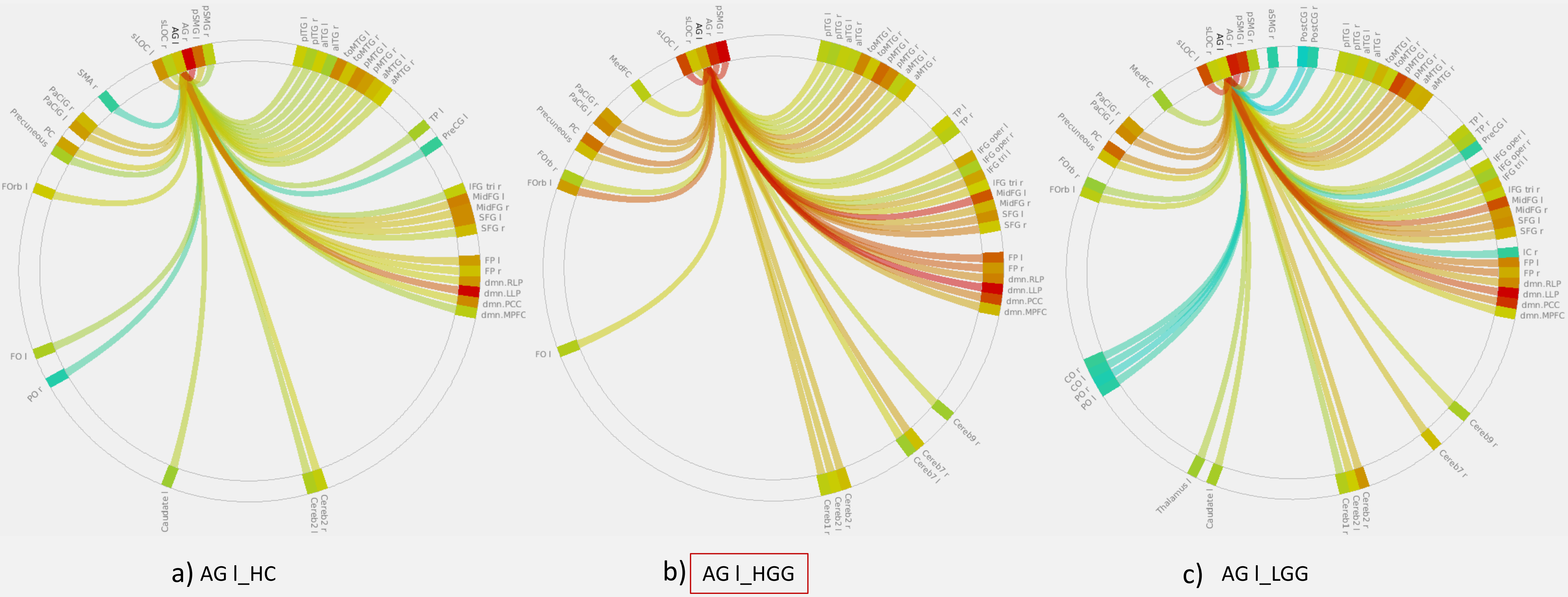

Whole-brain connectivity diagrams generated by seeding the left angular gyrus (AG l) in healthy controls (HC), left-hemispheric high-grade gliomas (HGG) and left-hemispheric low-grade gliomas (LGG). The red square in diagram b) indicates that there was a significant difference in functional connectivity in this seed for HGG compared to HC. The color scale of the links represents their statistical significance (p-value FDR corrected, only links with $p<0.05$ are included). Regions of interest (ROI) in the diagrams are labeled as per the AAL atlas (available in CONN toolbox at https://web.conn-toolbox.org)

Figure S16. Whole-brain connectivity diagrams of the left insular cortex

Higher p — Lower p

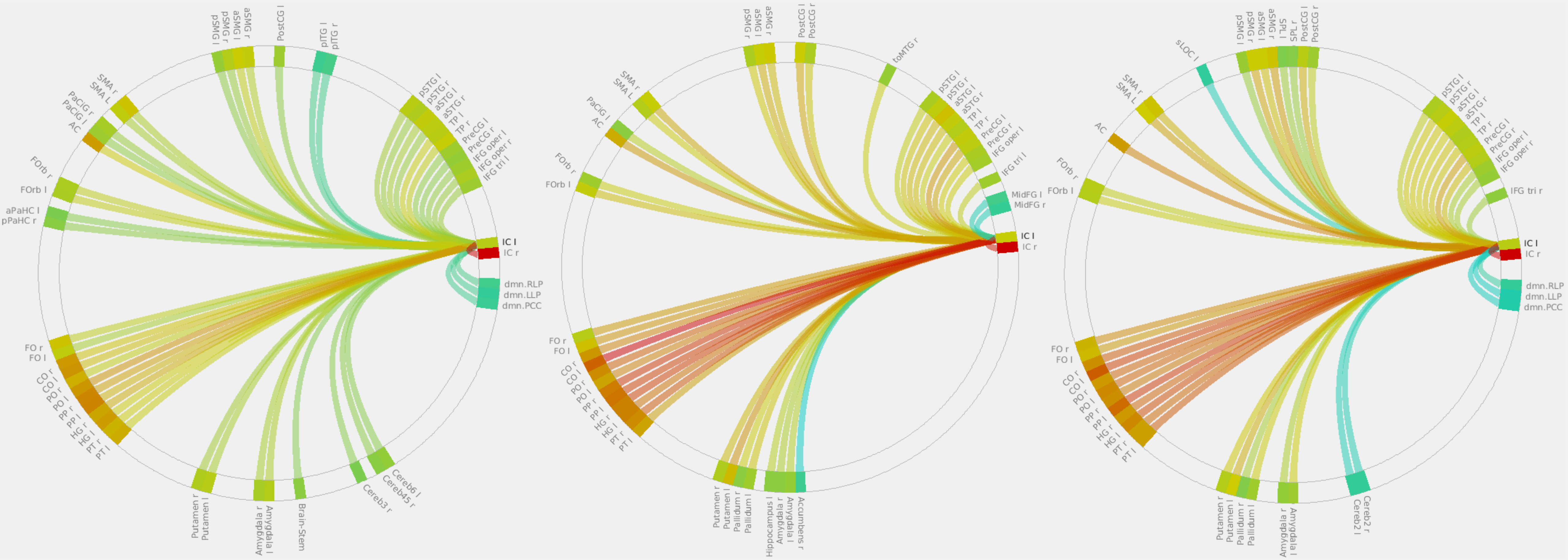

a) IC l_HC b) IC l_HGG c) IC l_LGG

Whole-brain connectivity diagrams generated by seeding the left insular cortex (IC l) in healthy controls (HC), left-hemispheric high-grade gliomas (HGG) and left-hemispheric low-grade gliomas (LGG). The red square in diagram c) indicates that there was a significant difference in functional connectivity in this seed for LGG compared to HC. The color scale of the links represents their statistical significance (p-value FDR corrected, only links with $p<0.05$ are included). Regions of interest (ROI) in the diagrams are labeled as per the AAL atlas (available in CONN toolbox at https://web.conn-toolbox.org)

Table S1. Summary of graph-theoretical metrics investigated in this study (available at https://web.conn-toolbox.org)

Degree	Cost	Average Path Length	Clustering Coefficient	Global Efficiency	Local Efficiency	Betweenness Centrality
Degree is defined at each node as the number of edges from/to each node. Degree at each node/ROI represents a measure of network centrality, characterizing the degree of local connectedness of each ROI within a graph. Similarly, network degree represent the average or the degree across all nodes within a graph.	Cost is defined at each node as the proportion of edges from/to each node. Cost at each node/ROI represents a measure of network centrality, characterizing the degree of local connectedness of each ROI within a graph. Similarly, network cost represent the average or the cost across all nodes within a graph.	Path length between each pair of nodes in a graph is defined as the minimum number of edges traversed in an optimal path between them. Average path length represents a measure of node centrality within a network, characterizing the degree of global connectedness of each ROI within a graph.	Clustering Coefficient is defined as the proportion of connected edges in the local neighboring sub-graph for each node/ROI. Clustering coefficient represents a measure of local integration, characterizing the degree of inter-connectedness among all nodes within a node neighboring sub-graph. Similarly, network clustering coefficient represents a measure of network locality or coherence.	Global Efficiency at a node is defined as the average of inverse-distances between this node and all other nodes in the same graph. Global efficiency at a node represents a measure of this node centrality within the network, characterizing the degree of global connectedness of each ROI. Similarly, network global efficiency represents a measure of inter-connectedness or radius of the entire network.	Local Efficiency at each node is defined as the Global efficiency of the neighboring sub-graph of this node. Local efficiency represents a measure of local integration or coherence, characterizing the degree of inter-connectedness among all nodes within a node neighboring sub-graph. Similarly, network local efficiency represents a measure of local integration in a network.	Betweenness centrality represents an alternative measure of node centrality within a graph. It is defined as the proportion of times that a node is part of a shortest-path between any two pairs of nodes within a graph.

Table S2. Clinical and demographic data for HGG patients

GRADE	HAND	SEX	AGE	BNT	NEURO LANGUAGE OE preop	NEURO LANGUAGE OE postop
4	1	M	72		0	
4	0	F	79	24	1	0
4	1	M	61	45	1	0
4	1	M	68		0	1
4	1	F	58	56	0	0
3	0	M	24	48	1	0
4	1	M	68		0	0
4	1	M	66		1	1
4	1	M	63		0	0
4	1	M	58		0	0
3	1	M	35		0	0
3	1	M	38		0	0
4	1	F	70	55	0	0
4	0	M	54	60	0	1
4	1	M	73	25	1	1
4	1	F	65	53	0	1
4	1	M	63		0	1
4	1	M	64		1	1
4	1	M	69	37	1	1
4	1	M	62	54	1	0
3	1	M	40		0	0
4	1	M	71		0	1
4	1	F	73		0	0
3	1	M	77	57	0	0
4	1	F	73		0	1
4	1	M	65		1	1
4	1	F	39		0	0
4	1	M	50		0	0
4	1	M	63		0	0
4	1	F	88		1	1

HAND = handedness (0=left, 1=right); BNT = Boston Naming Test (60 elements); OE = objective examination (0=no aphasia, 1=aphasia)

Table S3. Clinical and demographic data for LGG patients

GRADE	HAND	SEX	AGE	BNT	NEURO LANGUAGE OE preop	NEURO LANGUAGE OE postop
2	1	M	28	57	0	0
2	1	M	50		0	0
2	0	F	38		0	1
2	1	M	45	54	0	0
2	1	M	34		0	0
2	1	M	40		0	0
2	1	M	22		0	0
2	1	F	66		0	0
2	1	M	33		0	0
2	1	M	46		0	0
2	1	F	22		0	0
2	1	M	31		0	0
2	1	F	39		0	0
2	1	M	28	57	0	1
2	1	M	28		0	0
2	1	M	46		0	0
2	1	F	38		0	0
2	1	F	49		0	0
2	0	M	60		0	1
2	0	M	36		0	0
2	1	F	33	57	0	
2	1	M	51		0	
2	0	F	50	59	0	
2	1	M	41		0	0
2	0	M	64	57	0	0
2	1	M	18	54	0	1
2	1	M	32	51	0	0
2	0	M	40		0	0
2	0	M	25		0	1
2	1	F	58	60	0	1

HAND = handedness (0=left, 1=right); BNT = Boston Naming Test (60 elements); OE = objective examination (0=no aphasia, 1=aphasia)

Table S4. Statistical description of tumor size and patients' age

	Tumor Size		Age	
	LGG	HGG	LGG	HGG
**Mean**	13.05	8.38	39.70	61.63
**Median**	12.01	6.58	38.50	64.50
**Mode**	7.00	4.32	28.00	63.00
**25%percentile**	7.61	4.32	31.00	58.00
**75%percentile**	16.57	11.56	48.00	71.00
**Standard Deviation**	7.08	5.94	12.55	14.33

Tumor size = cm^2; Patients' age = years

Table S5. Whole brain network mean values of graph-theory results

	Global Efficiency	Local Efficiency	Betweenness Centrality	Cost	AveragePath Length	Clustering Coefficient	Degree
HC	0.485714	0.718755	0.010047	0.139641	2.368438	0.515864	18.85147
HGG	0.490513	0.708368	0.009726	0.14061	2.326033	0.503357	18.98235
LGG	0.491249	0.715634	0.009796	0.140356	2.328269	0.510522	18.94804

Table S6. Left hemispheric network mean values of graph-theory results

	Globa lEfficiency	Local Efficiency	Betweenness Centrality	Cost	AveragePath Length	Clustering Coefficient	Degree
HC	0.423239	0.682214	0.031559	0.144286	2.645243	0.544371	7.07
HGG	0.42729	0.663264	0.031445	0.14302	2.628351	0.526015	7.008
LGG	0.441973	0.667678	0.033641	0.142503	2.678038	0.524488	6.982667

Table S7. Right hemispheric network mean values of graph-theory results

	Global Efficiency	Local Efficiency	Betweenness Centrality	Cost	AveragePath Length	Clustering Coefficient	Degree
HC	0.431523	0.675539	0.032204	0.147837	2.654064	0.541028	7.244
HGG	0.413517	0.687953	0.029934	0.142585	2.616491	0.555485	6.986667
LGG	0.427586	0.696122	0.032457	0.143837	2.678345	0.5555	7.048

Table S8. Mean values of significant graph-theory results for gliomas involving the frontal lobe

**SFG r**	Global Efficiency	Local Efficiency	Betweenness Centrality	Cost	AveragePath Length	Clustering Coefficient	Degree
HGG	0.477333	0.536602	0.010666	0.139259	2.446312	0.326347	18.8
LGG	0.549605	0.713216	0.016961	0.192098	2.071295	0.466454	25.93333
HC	0.495265	0.700928	0.010501	0.142592	2.329970	0.483163	19.25

SFG r = superior frontal gyrus right

Table S9. Mean values of significant graph-theory results for gliomas involving the frontal lobe

	**SFG l**	**MidFG l**	**IFG tri l**	**IFG tri l**
	AveragePath Length	Clustering Coefficient	Betweenness Centrality	Clustering Coefficient
HGG	2.442470	0.482575	0.018026	0.373210
LGG	2.173093	0.495546	0.007203	0.483435
HC	2.327395	0.428003	0.007744	0.514210

MidFG l = middle frontal gyrus left; SFG l = superior frontal gyrus left; IFG tri l = triangular part of the left inferior frontal gyrus

Table S10. Mean values of significant graph-theory results for gliomas involving the temporal lobe

	**aSTG l** Global Efficiency	**aSTG l** Cost	**aSTG l** Degree	**pSTG l** Global Efficiency	**pSTG l** Local Efficiency	**pSTG l** Cost	**pSTG l** Clustering Coefficient	**pSTG l** Degree
HGG	0.385247	0.137755	6.75	0.478742	0.670184	0.123148	0.429484	16.62
LGG	0.479465	0.121605	16.41	0.488796	0.664110	0.119135	0.457369	16.08
HC	0.540463	0.198889	26.85	0.531987	0.788925	0.202962	0.583620	27.4

aSTG l = superior temporal gyrus anterior division left; pSTG l = superior temporal gyrus posterior division left

Table S11. Mean values of significant graph-theory results for gliomas involving the temporal lobe

	**aMTG l**<br>Betweenness Centrality	**aMTG l**<br>Cost	**aMTG l**<br>Degree	**aITG l**<br>Cost	**aITG l**<br>Degree
HGG	0.025105	0.105867	5.1875	0.130101	6.375
LGG	0.004922	0.094444	12.75	0.087654	11.833333
HC	0.011448	0.171481	23.15	0.124074	16.75

aITG l = inferior temporal gyrus anterior division left; aMTG l = middle temporal gyrus anterior division left

Table S12. Mean values of significant graph-theory results for gliomas involving the temporal lobe

	**pSTG r**	**pMTG r**	**pMTG r**	**pMTG r**	**toMTG r**	**toMTG r**	**aITG r**
	Betweenness Centrality	Local Efficiency	Cost	Degree	Cost	Degree	Clustering Coefficient
HGG	0.007628	0.69154	0.163425	22.0625	0.15	20.25	0.49059
LGG	0.039555	0.651497	0.212345	28.666666	0.166666	8.166666	0.407299
HC	0.013726	0.739019	0.211851	28.6	0.205185	27.7	0.569916

pSTG r = superior temporal gyrus posterior division right; pMTG r = middle temporal gyrus posterior division right; toMTG r = middle temporal gyrus temporo-occipital division right; aITG r = inferior temporal gyrus anterior division right

Table S13. Mean values of significant graph-theory results for parietal gliomas

	**PostCG l**	**PostCG l**	**AG l**	**AG l**
	LocalEfficiency	BetweennessCentrality	GlobalEfficiency	AveragePathLength
HGG	0.703477	0.013985	0.538877	2.143081
LGG	0.712518	0.013083	0.495918	2.415228
HC	0.793269	0.00625	0.495160	2.341090

AG l = angular gyrus left; AV = average; HC = healthy controls; HGG = high-grade glioma; LGG = low-grade glioma; PostCG_l = post-central gyrus left

Table S14. Mean values of significant graph-theory results for insular gliomas

	**IC l**<br>Cost	**IC l**<br>Degree
HGG	0.142857	7
LGG	0.156173	21.083333
HC	0.208889	28.2

IC l = insular cortex left
